# Fortified balanced energy–protein supplementation during pregnancy and lactation and infant growth in rural Burkina Faso: A 2 × 2 factorial individually randomized controlled trial

**DOI:** 10.1371/journal.pmed.1004186

**Published:** 2023-02-06

**Authors:** Alemayehu Argaw, Brenda de Kok, Laeticia Celine Toe, Giles Hanley-Cook, Trenton Dailey-Chwalibóg, Moctar Ouédraogo, Anderson Compaoré, Katrien Vanslambrouck, Rasmané Ganaba, Patrick Kolsteren, Carl Lachat, Lieven Huybregts

**Affiliations:** 1 Department of Food Technology, Safety and Health, Faculty of Bioscience Engineering, Ghent University, Ghent, Belgium; 2 Department of Population and Family Health, Institute of Health, Jimma University, Jimma, Ethiopia; 3 Unité Nutrition et Maladies Métaboliques, Institut de Recherche en Sciences de la Santé (IRSS), Bobo-Dioulasso, Burkina Faso; 4 AFRICSanté, Bobo-Dioulasso, Burkina Faso; 5 Poverty, Health, and Nutrition Division, International Food Policy Research Institute (IFPRI), Washington, DC, United States of America

## Abstract

**Background:**

Optimal nutrition is crucial during the critical period of the first 1,000 days from conception to 2 years after birth. Prenatal and postnatal supplementation of mothers with multimicronutrient-fortified balanced energy–protein (BEP) supplements is a potential nutritional intervention. However, evidence on the long-term effects of BEP supplementation on child growth is inconsistent. We evaluated the efficacy of daily fortified BEP supplementation during pregnancy and lactation on infant growth in rural Burkina Faso.

**Methods and findings:**

A 2 × 2 factorial individually randomized controlled trial (MISAME-III) was implemented in 6 health center catchment areas in Houndé district under the Hauts-Bassins region. From October 2019 to December 2020, 1,897 pregnant women aged 15 to 40 years with gestational age <21 completed weeks were enrolled. Women were randomly assigned to the prenatal intervention arms receiving either fortified BEP supplements and iron–folic acid (IFA) tablets (i.e., intervention) or IFA alone (i.e., control), which is the standard of care during pregnancy. The same women were concurrently randomized to receive either of the postnatal intervention, which comprised fortified BEP supplementation during the first 6 months postpartum in combination with IFA for the first 6 weeks (i.e., intervention), or the postnatal control, which comprised IFA alone for 6 weeks postpartum (i.e., control). Supplements were provided by trained village-based project workers under direct observation during daily home visits. We previously reported the effect of prenatal BEP supplementation on birth outcomes. The primary postnatal study outcome was length-for-age z-score (LAZ) at 6 months of age. Secondary outcomes were anthropometric indices of growth (weight-for length and weight-for-age z-scores, and arm and head circumferences) and nutritional status (prevalence rates of stunting, wasting, underweight, anemia, and hemoglobin concentration) at 6 months. Additionally, the longitudinal prevalence of common childhood morbidities, incidence of wasting, number of months of exclusive breastfeeding, and trajectories of anthropometric indices from birth to 12 months were evaluated. Prenatal BEP supplementation resulted in a significantly higher LAZ (0.11 standard deviation (SD), 95% confidence interval (CI) [0.01 to 0.21], *p* = 0.032) and lower stunting prevalence (−3.18 percentage points (pp), 95% CI [−5.86 to −0.51], *p* = 0.020) at 6 months of age, whereas the postnatal BEP supplementation did not have statistically significant effects on LAZ or stunting at 6 months. On the other hand, postnatal BEP supplementation did modestly improve the rate of monthly LAZ increment during the first 12 months postpartum (0.01 z-score/month, 95% CI [0.00 to 0.02], *p* = 0.030), whereas no differences in growth trajectories were detected between the prenatal study arms. Furthermore, except for the trend towards a lower prevalence of underweight found for the prenatal BEP intervention at 6 months (−2.74 pp, 95% CI [−5.65 to 1.17], *p* = 0.065), no other secondary outcome was significantly affected by the pre- or postnatal BEP supplementation.

**Conclusions:**

This study provides evidence that the benefits obtained from prenatal BEP supplementation on size at birth are sustained during infancy in terms of linear growth. Maternal BEP supplementation during lactation may lead to a slightly better linear growth towards the second half of infancy. These findings suggest that BEP supplementation during pregnancy can contribute to the efforts to reduce the high burden of child growth faltering in low- and middle-income countries.

**Trial registration:**

ClinicalTrials.gov: NCT03533712.

## Abstract 2

Carl Lachat and colleagues report a a 2×2 factorial individually randomized controlled trial conducted in rural Burkina Faso which investigates the impact of fortified balanced energy-protein supplementation during pregnancy and lactation on infant growth.

## Author summary

**Why was this study done?:**

- Growth faltering during the first 1,000 days is a global public health problem, which is strongly associated with an increased risk of child mortality and poor developmental outcomes, as well as poor school performance and lower earning in later life.
- Prenatal and postnatal supplementation of mothers with multiple micronutrient-fortified balanced energy–protein (BEP) supplements is a potential nutritional intervention to prevent the high burden of growth retardation during this window of opportunity in low- and middle-income countries.
- However, the effect of providing pregnant and lactating women with BEP supplements on child growth is inconsistent to date. Hence, there is a critical need for high-quality experimental studies to assess the efficacy of BEP supplements on fetal and infant growth outcomes.

**What did the researchers do and find?:**

- We conducted an individually randomised controlled efficacy trial in rural Burkina Faso (MISAME-III) to assess the effect of supplementing mothers with fortified BEP during pregnancy and lactation on the growth of their infants.
- Our trial indicates that the improvements in size at birth accrued from prenatal BEP supplementation are sustained in terms of linear growth at the age of 6 months.
- In addition, we provide evidence that maternal BEP supplementation for 6 months postpartum might lead to a slightly better linear growth towards the second half of infancy.

**What do these findings mean?:**

- Our findings suggest that the benefits of BEP supplementation on linear growth might be fully exploited by extending the intervention to cover the first 1,000 days of life, which includes supplementation of infants and young children during the complementary feeding period.
- Future research in MISAME-III will assess additional maternal and child biochemical parameters to provide further insights into the clinical relevance of BEP supplementation, including the potential impacts on breast milk composition.

## Introduction

According to the 2021 global burden of malnutrition estimates, 149.2 million (22%) children under the age of 5 suffer from stunted growth and may never develop to their full potential [1]. Child stunting, defined as length-for-age z-score (LAZ) below 2 standard deviations (SDs) from the World Health Organization (WHO) Child Growth Standard median, is associated with a higher susceptibility to illness and mortality, and there is growing evidence that children with stunting continue to perform less well in school and earn less as adults [2,3]. Stunted growth often begins in utero and typically aggravates during infancy until the age of 18 to 23 months and after 2 years of age, the faltering in growth is largely irreversible [4–6]. It is therefore important to focus nutritional interventions on the crucial period of pregnancy and the first 2 years of life, i.e., the first 1,000 days, to promote optimal growth [4].

WHO recommends exclusive breastfeeding (EBF) for the first 6 months of life [7]. During this period, EBF is expected to provide all the necessary nutrients and bioactive compounds to promote optimal child health, growth, and development [8]. While human breast milk volume and protein content is largely unaffected by maternal nutritional status [9,10], the quality of fatty acids is found to be correlated with maternal dietary intakes [11]. In addition, mothers’ dietary patterns can affect the quantity of micronutrients in breast milk, mainly water-soluble vitamins and trace minerals, such as selenium and iodine [8,10,12]. In sub-Saharan Africa, promotion of EBF alone has limited impact on improving linear growth of infants [13]. Providing nutritional support to lactating women therefore may have the potential to improve maternal status and prevent child malnutrition during the earliest periods of life.

The evidence on the effect of providing pregnant and lactating women with lipid-based nutrient supplements (LNS) on child growth is inconsistent to date. Randomized controlled trials (RCTs) in Malawi [14], Niger [15], and Burkina Faso [16] assessing the effect of prenatal small-, medium-, and large-quantity LNS supplementation, respectively, did not indicate effects on LAZ or stunting at 3, 12, or 24 months of age. On the other hand, a multicountry study conducted in the Democratic Republic of Congo, Guatemala, India, and Pakistan found that preconception and prenatal small-quantity LNS led to greater child linear growth at 6 months in 3 out of 4 countries and a lower relative risk for stunting [17]. Other RCTs evaluated the effect of continued maternal LNS supplementation during lactation and, in some cases, extended supplementation to infants from the age of 6 months onwards. Two of these studies, in Ghana [18] and Bangladesh [19], showed a positive effect of small-quantity LNS on LAZ and stunting prevalence at the age of 18 months. On the contrary, studies in Malawi [20,21], Guatemala [22], Madagascar [23], and Bangladesh [24] did not find any effect of LNS supplementation during pregnancy and lactation on LAZ or stunting in the full sample. In response to the mixed results of LNS supplementation, with often modest effect sizes, additional evidence based on high-quality individually randomized trial is needed on the impact of maternal fortified balanced energy–protein (BEP) supplementation on infant growth. Furthermore, to our knowledge, there are no studies that evaluated the interaction between prenatal and postnatal maternal BEP supplementation on child linear growth and nutritional status. Yet, it is essential to understand the added value of continued BEP supplementation after delivery and what unique contribution to infant’s growth and nutritional status it represents as compared to prenatal BEP.

BEP supplements are a similar type of ready-to-use supplements that provide multiple micronutrients and, specifically, energy and protein in a balanced composition (<25% of total kcal content from protein) to address maternal malnutrition during pregnancy and lactation. We previously reported on the modest efficacy of prenatal BEP supplementation on weight and length increments at birth and the lower prevalence of low birth weight in the MIcronutriments pour la SAnté de la Mere et de l’Enfant-III (MISAME-III) trial [25]. The objective of the current study was to evaluate the efficacy of BEP supplementation during 6 months postpartum on infant growth and nutritional status, to assess possible interactions between prenatal and postnatal BEP supplementation, and to investigate whether the effects of the prenatal BEP supplementation persist at the age of 6 months.

## Methods

Our research was reported using the Consolidated Standards of Reporting Trials (CONSORT) 2010 checklist **[26].**

### Study setting and design

The MISAME-III study protocol has been described elsewhere in detail [27]. In brief, MISAME-III is an individually RCT evaluating the efficacy of a combination of a daily fortified BEP supplement and iron–folic acid (IFA) tablet, as compared to IFA supplementation alone, during pregnancy and lactation. The study follows a 2 × 2 factorial design where women were enrolled into the study to receive BEP supplementation during pregnancy (prenatal intervention) and/or the first 6 months of lactation (postnatal intervention). Therefore, women were randomized into one of the 4 study groups: (1) prenatal BEP and IFA supplementation; (2) postnatal BEP and IFA supplementation; (3) both pre- and postnatal BEP and IFA supplementation; or (4) both pre- and postnatal IFA only supplementation.

The study was implemented in 6 rural health center catchment areas in the district of Houndé in the Hauts-Bassins region of Burkina Faso. The study area is characterized by a Sudano-Sahelian climate with a dry season running between September/October and April. Agricultural activity is the main livelihood in the study area, with dominantly cotton and maize production. The habitual diet during pregnancy is nondiverse [28], predominantly based on maize with a complement of leafy vegetables [29]. Hence, among a subsample of MISAME-III women, dietary micronutrient intakes of the base diet (i.e., excluding supplements) during pregnancy were inadequate to cover the Estimated Average Requirements (EARs) and the mean daily energy intake was estimated to be approximately 1,940 kcal at the end of the preharvest season [30]. Furthermore, findings from MISAME-III indicate high burden of adverse birth outcomes, including small-for-gestational age (26%) and low birthweight (10%) [25]. In addition, a survey in the same region found a high prevalence of child stunting (21%) and wasting (7%), whereas the rate of EBF was reported to be 62% [31].

Before MISAME-III commenced, women aged between 15 and 40 years living in the study area were identified through a door-to-door census (*n =* 10,165). Between October 2019 and December 2020, a network of female village workers trained for the project conducted follow-up home visits of these women every 5 weeks for early identification of pregnancy through self-reported amenorrhea. Women who reported amenorrhea were referred to the nearest health center for a urinary pregnancy test and their first antenatal care consultation. Following a positive test result, women were asked for their informed written consent to participate in the study and randomized into the pre- and postnatal intervention arms. Within 14 days of enrollment, pregnancy was confirmed and gestational age was determined by ultrasonography. Study exclusion criteria were nonconfirmed pregnancies by ultrasound, pregnancies ≥21 completed weeks of gestation at enrollment, or multifetal pregnancies (i.e., postrandomization exclusion). Furthermore, women who planned to leave or deliver outside the study area and women who reported to be allergic to peanuts were not enrolled. The MISAME-III study was approved by the ethics committee of the University Hospital of Gent, Belgium and the ethics committee of Centre Muraz, Burkina Faso. The trial is registered at ClinicalTrials.gov with registration number NCT03533712.

### Randomization

A stratified permuted block randomization schedule was used to assign women to either the intervention or control arms of the pre- and postnatal interventions. Randomization blocks were generated per health center in blocks of 8 (i.e., 4 controls and 4 interventions arms for each of the pre- and postnatal intervention) using Stata 15.1 (Statacorp, Texas, USA) by a research analyst not involved in the study. The allocation was coded with the letters A (control) or B (intervention) for the prenatal intervention arms, and X (control) and Y (intervention) for the postnatal intervention arms. The allocation letter codes were concealed in sequentially numbered separate sealed opaque envelops by the study employees who had no direct contact with the study participants. At enrolment during the first antenatal visit, study midwives assigned the women to the study groups by drawing the next sealed envelope with the letter code.

### Intervention and procedures

The composition and daily dose of the pre- and postnatal BEP intervention supplements were identical; only the duration of supplementation differed. The BEP supplement, an LNS in the form of an energy-dense peanut paste fortified with multiple micronutrients, was selected based on a two-phase formative study conducted in the same study community [32,33]. The complete nutritional composition of a daily dose of the fortified BEP is provided in S1 Table. The product is ready to use, does not require a cold chain, and is highly stable with a long shelf life. A daily dose of the BEP (72 g) provided an energy top up of 393 kcal (36% energy from lipids, 20% from protein, and 32% from carbohydrates) and covered at least the EARs of pregnant women for 11 micronutrients, except calcium, phosphorous, and magnesium [34]. A daily dose of an IFA tablet (Sidhaant Life Sciences, Delhi, India) contained 65 mg of iron [form: FeH_2_O_5_S] and 400 μg folic acid [form: C_19_H_19_N_7_O_6_], in accordance with the standard of care in Burkina Faso. The prenatal intervention provided either a daily BEP supplement plus IFA tablet (intervention) or IFA alone (control) starting from enrollment, during the first/early second trimester (average gestational age of 11.8 weeks) to birth. In the postnatal intervention, BEP was provided to women in the intervention group for 6 months starting from birth. Women in the intervention and control group both received IFA tablets for 6 weeks postpartum.

Once enrolled, women were assigned to the study’s village-based project workers, who were responsible for conducting daily home visits to provide the supplements and conduct compliance assessment through direct observation of supplement intake. During these visits, pregnant women were encouraged to attend all antenatal care visits, give birth at the health center, and attend monthly postnatal health center visits. Furthermore, the importance of a healthy diet during pregnancy, as well as the importance of optimal infant feeding practices, was discussed. Supervision of village-based workers was conducted on monthly bases throughout the study using a Lot Quality Assurance Sampling scheme and empty sachet counts to ensure that study participants were visited according to the project protocol.

After delivery, mothers were invited to attend monthly growth monitoring sessions organized by study midwives at their nearest health center until all study children reached the age of 6 months. Subsample of children who were born earlier in the study cohort had a continued followed-up through home visits at approximately 9 months (*n =* 1,345) and approximately 12 months (*n* = 955) of age. During the growth monitoring sessions, midwives counselled the mother on EBF practices and carried out infant anthropometry measurements. Infant weight was measured with a digital Seca 384 scale to the nearest 10 gm, whereas length was measured with Seca 416 length board to the nearest 1 mm. Head and mid-upper arm circumference (MUAC) measurements were taken to the nearest 1 mm with a Seca 212 measuring tape. Measurements were taken in duplicates, and when there is a large discrepancy between the duplicate measures (>10 mm for length, >200 g for weight, and >5 mm for head and arm circumferences), a third measurement was carried out, and, finally, the average of the two closest measurements was used for analysis. The occurrence of common childhood morbidities (fever, diarrhea, vomiting, runny nose, cough, difficulty of breathing, grunting, and skin lesions) was assessed by asking mothers how many days over the past 7 days their infant experienced each of these morbidity symptoms. Diarrhea was defined as the occurrence of ≥3 liquid or semisolid stools within a day. Furthermore, armpit body temperature measurements were taken by study midwives, and fever was diagnosed as a body temperature ≥37.5°C. At the age of 6 months, infant hemoglobin concentration (g/dL) was determined from a finger prick capillary blood sample using HemoCue Hb 201+ instrument. Information about infant feeding practices was also gathered by maternal 24-hour recall during the monthly anthropometric measurements. Duration of EBF was estimated by asking mothers the date the infant started receiving liquid/solid foods. Minimum dietary diversity for children was assessed at 9 and 12 months of age and achieved when an infant consumed foods and beverages from at least 5 out of 8 defined food groups during the previous day (i.e., (i) breast milk; (ii) grains, roots, tubers, and plantains; (iii) pulses (beans, peas, lentils), nuts, and seeds; (iv) dairy products (milk, infant formula, yogurt, cheese); (v) flesh foods (meat, fish, poultry, organ meats); (vi) eggs; (vii) vitamin A–rich fruits and vegetables; and (viii) other fruits and vegetables) [35]. Infants with acute illnesses and acute malnutrition were referred to the nearest health center.

### Outcomes

The primary outcome of this study was children’s LAZ at the age of 6 months. Secondary outcomes included a suite of indices of growth and nutritional status at the age of 6 months, such as weight-for-length (WLZ) and weight-for-age (WAZ) z-scores, MUAC and head circumference, prevalence rates of stunting (LAZ <−2 SD), wasting (WLZ <−2 SD), and underweight (WAZ <−2 SD), hemoglobin concentration, and anemia (<11 g/dL). Additional secondary outcomes included the longitudinal prevalence of common childhood morbidities and the incidence of wasting and EBF during the 6 months follow-up. Furthermore, growth trajectories of infants were considered as additional secondary outcomes by evaluating monthly changes in the aforementioned anthropometric indices during the 12 months follow-up period. We previously reported the primary and secondary outcomes at birth [25].

### Statistical analysis

In the absence of dependency between the effects of the pre- and postnatal interventions (see below), a sample size of 588 children per study arm (*n =* 1,176) was required to detect a difference of 0.18 z-score (with an SD of 1.1) in LAZ between study arms at the age of 6 months with an 80% power and a 95% confidence level [36]. A total of 1,891 pregnant women were required to accommodate for a potential 26% loss of information during the prenatal period due to a combination of abortions, miscarriages, stillbirths, multifetal pregnancies, out-migrations, maternal deaths and incomplete data as detected in the previous MISAME trial [37], and a further anticipated 16% lost-to-follow-up from birth to 6 months postpartum.

All analyses in this study were documented a priori in the MISAME-III statistical analysis plan, which was published in the study website on November 3, 2020 (https://www.misame3.ugent.be/resource-files/MISAME-III_SAP_v1_102019.pdf). Analyses were conducted using Stata 17.1 (Statacorp, Texas, USA) and a two-sided statistical significance was considered at alpha <0.05. Descriptive statistics are reported as means ± SD for the continuous variables and as percentages for the nominal variables. Anthropometric indices of LAZ, WLZ, and WAZ were calculated based on the WHO 2006 Child Growth Standards [38].

The analysis strategy to evaluate the effects of BEP supplementation on infant outcomes was established based on the presence or absence of dependency (i.e., statistically significant interaction at *p* < 0.1) between the effects of the pre- and postnatal interventions [39]. Accordingly, due to the lack of a statistically significant interaction on the study outcomes (e.g., LAZ p-interaction = 0.767), we followed a factorial approach evaluating the two interventions separately. In this approach, the effect of each of the pre- and postnatal interventions on the study outcomes were evaluated independently of the effect of the other intervention (i.e., estimating the effect of one intervention by adjusting for the allocation to the other intervention).

Group differences in growth and nutritional status of infants at the age of 6 months (also at 9 and 12 months in a subsample) were estimated by fitting linear regression models for the continuous outcomes and linear probability models with robust variance estimation for the binary outcomes. Poisson regression models with robust variance estimation were fitted to estimate risk ratios between study arms in terms of the number of months infants received EBF and the number of months infants were diagnosed with wasting during the 6 months postpartum. All models contained the health center and randomization block as fixed effects to account for any possible clustering by the study design. Adjusted models further included prespecified known prognostic factors of the study outcomes such as maternal age, height, body mass index (BMI), MUAC, hemoglobin concentration, gestational age, and parity at study enrollment. Our main analysis followed a modified intention-to-treat (mITT) principle in which all infants who had birth anthropometry measurements were included in the analyses at 6 months. For this purpose, we conducted multiple imputations of missing data using chained equations under the “missing-at-random assumption.” Fifty imputations of missing data were conducted to estimate the regression coefficients. We further assessed the robustness of our findings by conducting sensitivity analyses such as a complete cases analysis (i.e., by including only infants with available measurements at 6 months) and a per-protocol analysis (i.e., by including only participants with BEP adherence of at least 75%).

As a secondary analysis, we modelled growth trajectories of infants from birth to 6 months, which continued up to 9 and 12 months on a subsample of infants. Mixed effects models with a random intercept for the individual infant and a random slope for the age of the child (in months) were fitted to estimate group differences on average monthly changes in LAZ, WLZ, WAZ, MUAC, and head circumference. We explored the best model fit for our data by assessing different potential relationships of the study outcomes with time, by visual inspection of graphs and comparing model fit indices (AIC and BIC). We applied linear models (WLZ and WAZ), quadratic model (LAZ), and restricted cubic spline models with 6 knots (MUAC and head circumference) according to the model fit. Fixed effects in the model included the main effect of group, time, and group by time interaction, which the later estimates difference between groups on monthly changes in the outcomes. All models included the clustering indicators (i.e., health center and randomization block), whereas adjusted models additionally included the aforementioned known maternal prognostic factors of infant growth.

Furthermore, we explored potential intervention effect modification on the primary outcome LAZ at 6 months by prespecified subgroup factors, such as maternal BMI (<18.5 kg/m^2^), MUAC (<23 cm), hemoglobin (<11 g/dl), height (<155 cm), age (<20 years), completion of primary education, possible and probable prenatal depression (Edinburgh Postnatal Depression Scale ≥10 points and ≥13 points), primiparity, household food insecurity (Household Food Insecurity Access Scale), newborn sex, season of delivery (lean season: June to September), and interpregnancy interval (<18 months).

The longitudinal prevalence rates of common childhood morbidities during the 6 months follow-up were calculated using “the total number of days that the outcome was reported” as numerator, and “the total days observed or assessed” as denominator. We used Poisson regression models with robust variance estimation to estimate risk ratios comparing study arms by the occurrence of morbidity outcomes.

Finally, to inform on the potential effect of the combination of the pre- and postnatal BEP interventions, we conducted supplementary analysis using a four intervention arms approach evaluating LAZ and stunting at 6 months and LAZ trajectories from birth to 12 months comparing the control group for both intervention periods (IFA/IFA) against each of the prenatal only BEP (BEP/IFA), the postnatal only BEP (IFA/BEP), and the combination of the pre- and postnatal BEP (BEP/BEP) groups.

### Inclusivity in global research

Additional information regarding the ethical, cultural, and scientific considerations specific to inclusivity in global research is included in S1 Supporting Information.

## Results

### Subject characteristics and study follow-up

**Fig 1:**
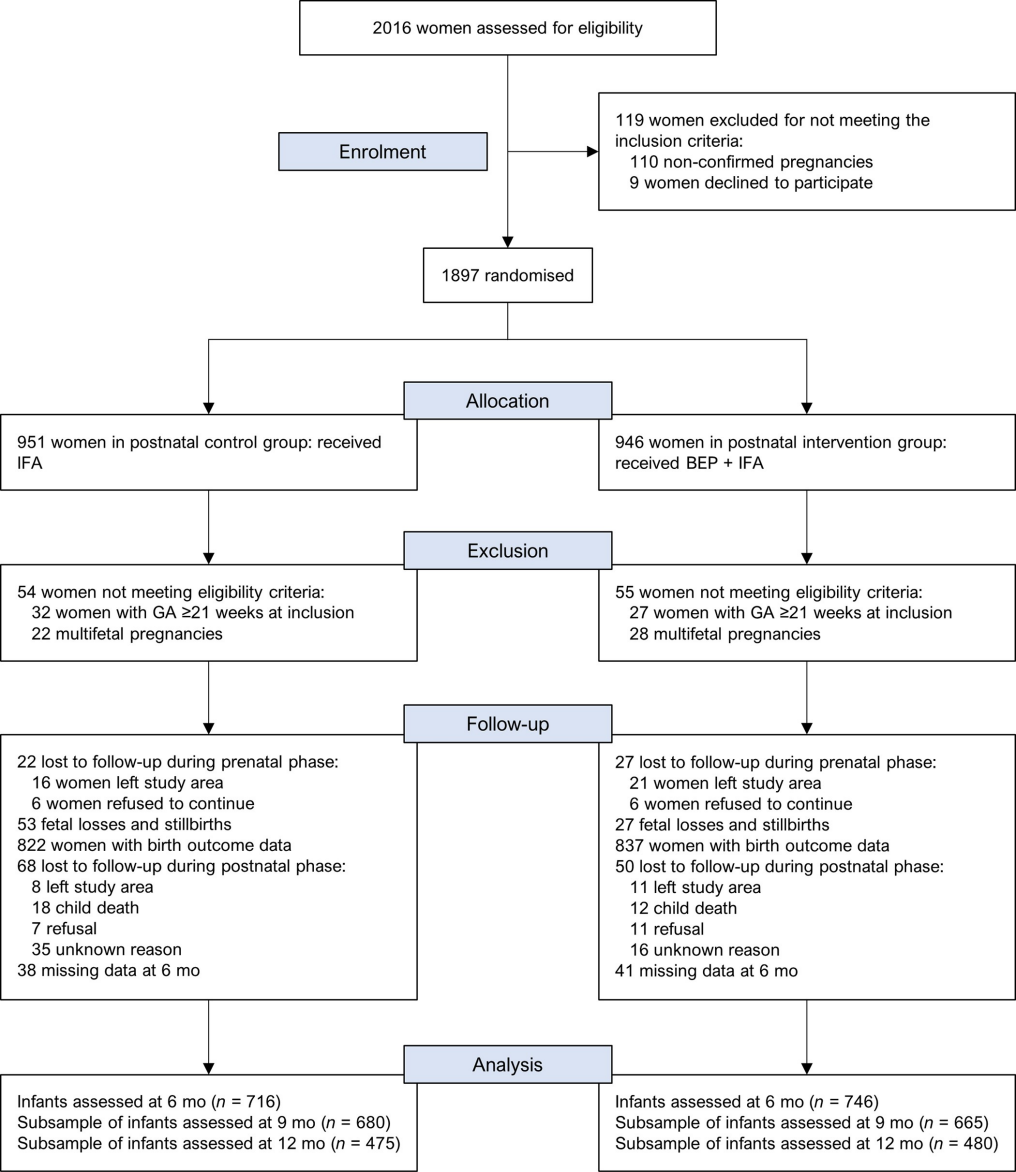
Trial flowchart of the MISAME-III study by the postnatal intervention arms. BEP, balanced energy–protein supplementation; GA, gestational age; IFA, iron–folic acid tablets; MISAME, MIcronutriments pour la SAnté de la Mère et de l’Enfant.

From a total of 2,016 women assessed for eligibility, 1,897 women were randomized into either the control or intervention arms of the pre- and postnatal interventions (Figs 1 and S1). Pregnancy was not confirmed for 110 women and 9 women refused to participate in the study. Postrandomization, an additional 109 women were excluded from the study due to multifetal pregnancies (*n =* 50) and gestational age ≥21 completed weeks at inclusion (*n* = 59) as a result of the ultrasound examination. A total of 1,659 newborns with birth anthropometry were identified, which were considered for the intention-to-treat analysis at 6 months. From this sample, anthropometry measurements were taken from 1,462 (88.1%), 1,345 (81.1%), and 955 (57.6%) infants at the age of 6, approximately 9, and approximately 12 months, respectively.

Baseline characteristics of study participants are presented in Table 1. Study arms for both the pre- and postnatal interventions were balanced with regard to maternal and household characteristics. Mean (SD) age of the study women was 25.0 (6.19) years and about 42.9% had at least 3 prior pregnancies. More than half of the mothers were from food insecure households, 7.11% were underweight, and 37.7% were anemic. Almost all infants were exclusively breastfed with an average duration of 5.61 months. Complementary feeding practices, however, were found to be very poor, with only 6.68% and 10.6% of infants meeting the minimum dietary diversity score at the age of 9 and 12 months, respectively (S2 Table). Overall, breastfeeding and complementary feeding practices remained comparable between the control and intervention arms for both the pre- and postnatal interventions. The average compliance rate of consuming the BEP supplement was 83.1% during the prenatal and 86.0% during the postnatal supplementation periods. Adherence to IFA tablets during pregnancy was 88.8% and 90.8% in the control and intervention arms, respectively. During the 6 weeks postpartum, adherence to IFA was 91.0% in the control and 88.4% in the intervention arm.

**Table 1:** Baseline characteristics of study participants by post- and prenatal intervention arms ^1^.

Characteristics	Postnatal intervention		Prenatal intervention	
	Control (*n* = 897)	Intervention (*n* = 891)	Control (*n* = 909)	Intervention (*n* = 879)
**Health center catchment area**				
Boni	22.3	21.5	22.0	21.8
Dohoun	10.3	11.2	10.5	11.0
Dougoumato II	17.9	18.5	18.9	17.5
Karaba	10.4	10.5	10.2	10.7
Kari	18.6	18.4	18.4)	18.7
Koumbia	20.5	19.8	20.0	20.3
**Household level**				
Wealth index, 0 to 10 points	4.58 ± 1.73	4.59 ± 1.77	4.51 ± 1.74	4.67 ± 1.75
Household food insecurity^2^	54.8	54.5	53.9	55.5
Improved primary water source^3^	62.5	62.3	62.2	62.7
Improved sanitation facility^4^	59.5	60.4	59.3	60.6
Household size	6.28 ± 4.30	6.11 ± 4.36	6.19 ± 4.45	6.20 ± 4.21
Polygamous households	33.9	30.5	31.8	32.7
**Maternal**				
Age, years	25.1 ± 6.07	25.0 ± 6.30	25.1 ± 6.20	25.0 ± 6.18
Ethnic group				
Bwaba	58.2	56.7	57.3	57.6
Mossi	34.4	35.4	35.3	34.5
Others	7.36	7.97	7.37	7.96
Religion pregnant woman				
Muslim	41.8	42.6	42.1	42.3
Animist	24.1	22.1	23.4	22.8
Protestant	16.1	18.5	16.2	18.4
Catholic	13.8	13.7	14.4	13.1
No religion, no animist	4.24	3.03	3.85	3.41
Primary education and above	40.5	43.3	42.4	41.4
Gestational age, weeks	11.6 ± 4.09	11.3 ± 4.03	11.4 ± 4.08	11.5 ± 4.04
Trimester of gestation				
First	61.9	63.3	63.1	62.0
Second	38.1	36.7	36.9	38.0
Parity				
0	21.2	23.7	21.8	23.1
1–2	33.9	35.5	35.9	33.4
3 or more	44.9	40.9	42.4	43.5
Weight, kg	58.1 ± 8.74	58.2 ± 8.61	57.9 ± 8.65	58.4 ± 8.69
Height, cm^5^	162 ± 6.21	163 ± 5.74	162 ± 5.91	163 ± 6.05
BMI, kg/m^2^	22.0 ± 2.86	21.9 ± 2.88	22.0 ± 2.87	22.0 ± 2.87
Mid-upper arm circumference, mm	263 ± 27.3	262 ± 25.9	262 ± 26.8	262 ± 26.4
Subscapular skinfold thickness, mm	11.9 ± 5.61	12.0 ± 5.44	11.9 ± 5.47	12.1 ± 5.58
Tripital skinfold thickness, mm	11.9 ± 4.89	11.9 ± 4.73	11.8 ± 4.76	12.0 ± 4.86
Hb, g/dL	11.3 ± 1.49	11.4 ± 1.50	11.4 ± 1.47	11.3 ± 1.52
Anemia, Hb <11 g/dL	38.2	37.1	36.7	38.7

^1^Values are percentages or means ± SDs.

^2^Assessed using FANTA/USAID’s Household Food Insecurity Access Scale.

^3^Protected well, borehole, pipe, or bottled water were considered improved water sources.

^4^Flush toilet connected to local sewage or septic tank, or pit latrine with slab and/or ventilation were considered improved sanitation facilities.

^5^Height of one woman with a physical disability could not be measured.

BMI, body mass index; Hb, hemoglobin; SD, standard deviation.

### Linear growth: Length-for-age z-score and stunting

**Fig 2:**
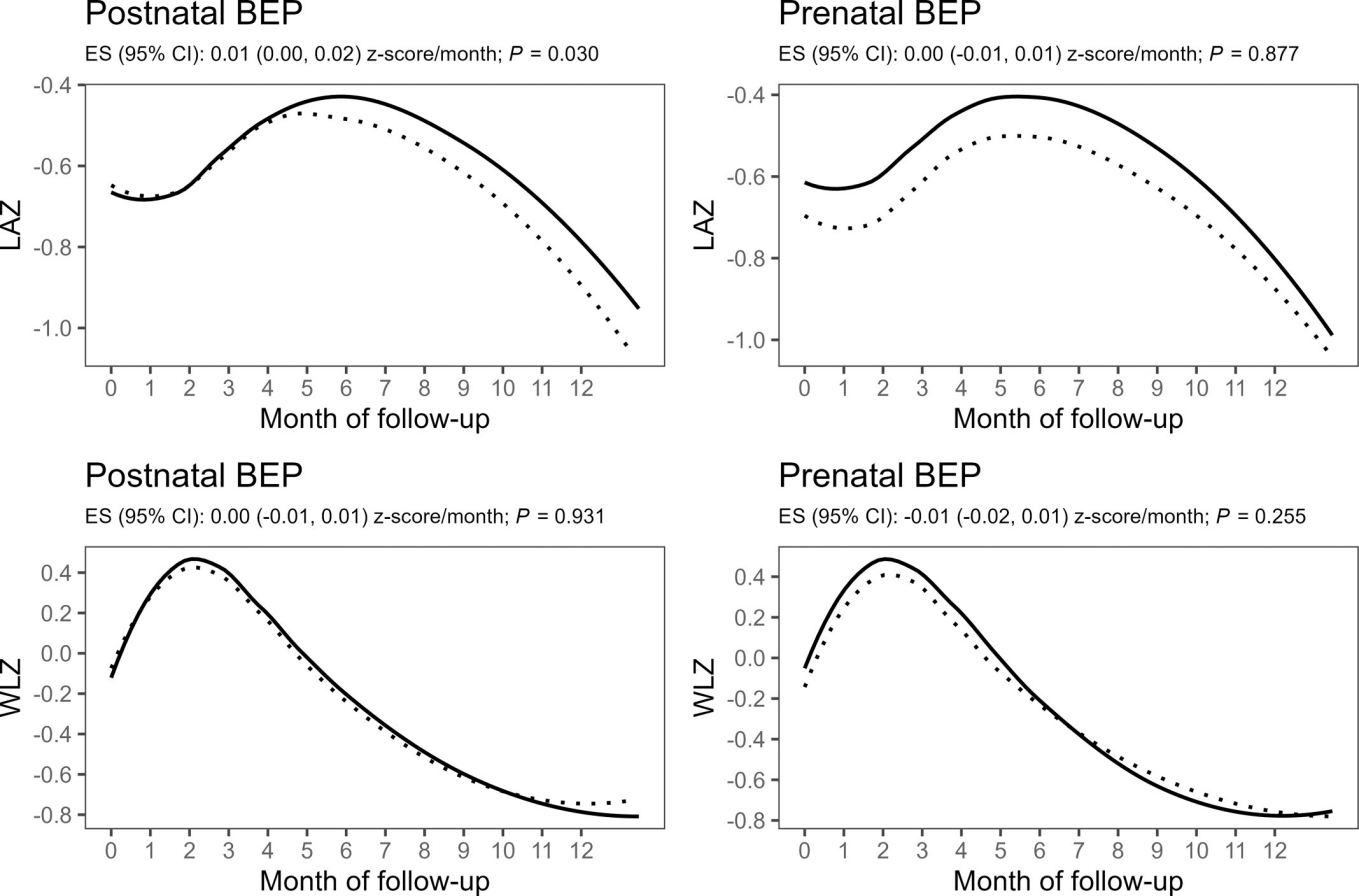
Monthly changes in LAZ (upper panels) and WLZ (lower panels) by postnatal (left panels) and prenatal (right panels) intervention arms with the dashed lines representing the control group (IFA) and solid lines representing the intervention group (IFA + BEP). Line graphs represent locally weighted scatterplot smoothing of observed values. Group differences were estimated using mixed-effects models with random intercepts for infant and random slopes for intervention time, with fixed effects including time, quadratic time (for LAZ), intervention group, and time × group interaction adjusted for allocation to the other intervention, clustering indicators (health center and randomization block), and a priori determined prognostic factors (maternal height, BMI, MUAC, hemoglobin, age and gestational age at inclusion, and parity). BEP, balanced energy–protein supplement; BMI, body mass index; CI, confidence interval; ES, regression coefficient; IFA, iron–folic acid; LAZ, length-for-age Z-score; MUAC, mid-upper arm circumference; WLZ, weight-for-length Z-score.

Postnatal BEP supplementation during the first 6 months postpartum did not result in a significant improvement in LAZ at the age of 6 months, our primary outcome (Table 2). On the other hand, BEP supplementation during pregnancy led to a modest, statistically significant increase in LAZ at the age of 6 months (0.11 SD, 95% confidence interval (CI) [0.01 to 0.21], *p* = 0.032) (Table 3). Similarly, prenatal BEP supplementation resulted in a 3.18 percentage points (pp) reduction in stunting prevalence at 6 months postpartum (95% CI [−5.86 to −0.51], *p* = 0.020), whereas no significant effect of the postnatal BEP supplementation was found on stunting prevalence at 6 months. When assessing the trajectories in monthly LAZ from birth to 12 months (Fig 2), infants from mothers who received the postnatal BEP supplementation showed a modestly improved LAZ growth rate as compared to the control group (0.01 z-score/month, 95% CI [0.00 to 0.02], *p* = 0.030). The prenatal BEP intervention arms, however, followed parallel LAZ growth trajectories maintaining the better newborn length attained at birth. In a cross-sectional comparison at 9 and 12 months on a subsample of infants, there was no statistically significant group difference in LAZ and stunting prevalence for both the post- and prenatal interventions (S3 Table). Only stunting prevalence at 9 months was reduced marginally in the prenatal BEP arm (−2.91 pp, 95% CI [−5.85 to 0.02], *p* = 0.052), compared to the control. Furthermore, we did not find evidence for effect modification by the subgroup factors on the effect of the postnatal BEP supplementation on LAZ at 6 months (S8 Table). Efficacy of the prenatal BEP supplementation on LAZ at 6 month was found to be larger among female infants, infants with nonanemic mothers, and infants born during the food plenty season (S9 Table).

**Table 2:** Effect of maternal postnatal BEP supplementation on infant growth and nutritional status at 6 months^1^.

Outcomes	Control (*n =* 822)	Intervention (*n* = 837)	Unadjusted difference (95% CI)	*p*	Adjusted difference (95% CI)	*p*
LAZ^2^	−0.55 ± 1.05	−0.48 ± 1.04	0.07 (−0.04, 0.17)	0.223	0.05 (−0.05, 0.15)	0.322
WLZ^2^	−0.23 ± 1.20	−0.19 ± 1.20	0.05 (−0.07, 0.18)	0.398	0.05 (−0.07, 0.17)	0.418
WAZ^2^	−0.58 ± 1.14	−0.51 ± 1.12	0.08 (−0.03, 0.20)	0.160	0.07 (−0.04, 0.19)	0.206
Arm circumference, mm^2^	140 ± 11.9	141 ± 12.1	0.73 (−0.45, 1.90)	0.225	0.74 (−0.42, 1.90)	0.213
Head circumference, cm^2^	41.9 ± 1.64	42.0 ± 1.60	0.14 (−0.03, 0.31)	0.098	0.13 (−0.03, 0.30)	0.109
Hb, g/dL^2^	10.5 ± 1.37	10.4 ± 1.33	−0.09 (−0.23, 0.04)	0.165	−0.10 (−0.23, 0.04)	0.155
Stunting (LAZ <−2 SD), %^3^	7.83	6.89	−0.90 (−3.63, 1.82)	0.516	−0.87 (−3.55, 1.81)	0.524
Wasting (WLZ <−2 SD), %^3^	6.30	6.29	0.13 (−2.58, 2.32)	0.917	0.01 (−2.44, 2.45)	0.996
Underweight (WAZ <−2 SD), %^3^	9.48	8.33	−1.36 (−4.29, 1.57)	0.362	−1.18 (−4.08, 1.73)	0.427
Anemia, Hb <11 g/dL^3^	62.2	65.9	3.96 (−0.99, 8.91)	0.117	3.90 (−5.03, 4.87)	0.124
Number of months receiving EBF^4^	5.00 ± 1.52	5.04 ± 1.53	1.01 (0.98, 1.04)	0.544	1.01 (0.98, 1.04)	0.548
Number of months with wasting^4^	0.22 ± 0.71	0.20 ± 0.65	0.90 (0.66, 1.25)	0.540	0.89 (0.64, 1.22)	0.467

^1^Values are means ± SDs or percentages. At age 6 months, unadjusted and adjusted group differences were estimated by fitting linear regression models for the continuous outcomes^2^, to estimate the mean group difference, and using linear probability models with robust variance estimators for the binary outcomes^3^, to estimate risk difference in percentage points. For the outcomes EBF and wasting episodes during the 6 months follow-up, we fitted Poisson regression models with robust variance estimation to compare study groups by the number of months with the outcome adjusted for log number of months assessed^4^. All models contained allocation to the prenatal intervention, and health center and randomization block as fixed effect to account for clustering by the study design. Adjusted models additionally contained a priori determined set of maternal prognostic factors such as age, parity, gestational age, height, MUAC, BMI, and Hb level at study enrolment.

BEP, balanced energy–protein supplement; CI, confidence interval; EBF, exclusive breastfeeding; Hb, hemoglobin; LAZ, length-for-age z-score; MUAC, mid-upper arm circumference; SD, standard deviation; WAZ, weight-for-age z-score; WLZ, weight-for-length z-score.

**Table 3:** Effect of maternal prenatal BEP supplementation on infant growth and nutritional status at 6 months^1^.

Outcomes	Control (*n =* 850)	Intervention (*n* = 809)	Unadjusted difference (95% CI)	*p*	Adjusted difference (95% CI)	*p*
LAZ^2^	−0.58 ± 1.07	−0.45 ± 1.02	0.12 (0.02, 0.23)	0.021	0.11 (0.01, 0.21)	0.032
WLZ^2^	−0.20 ± 1.21	−0.22 ± 1.18	0.00 (−0.13, 0.12)	0.963	−0.01 (−0.13, 0.11)	0.871
WAZ^2^	−0.58 ± 1.16	−0.51 ± 1.10	0.08 (−0.04, 0.20)	0.183	0.07 (−0.05, 0.18)	0.254
Arm circumference, mm^2^	140 ± 12.2	141 ± 11.8	0.95 (−0.23, 2.14)	0.116	0.85 (−0.31, 2.02)	0.151
Head circumference, cm^2^	41.8 ± 1.73	42.0 ± 1.49	0.15 (−0.02, 0.31)	0.091	0.14 (−0.02, 0.30)	0.091
Hb, g/dL^2^	10.4 ± 1.36	10.4 ± 1.34	0.01 (−0.12, 0.14)	0.835	0.02 (−0.11, 0.15)	0.811
Stunting (LAZ <−2 SD), %^3^	8.97	5.65	−3.32 (−6.04, −0.61)	0.017	−3.18 (−5.86, −0.51)	0.020
Wasting (WLZ <−2 SD), %^3^	6.41	6.17	−0.43 (−2.91, 2.06)	0.737	−0.31 (−2.78, 2.16)	0.806
Underweight (WAZ <−2 SD), %^3^	10.3	7.45	−3.03 (−5.98, −0.08)	0.044	−2.74 (−5.65, 1.17)	0.065
Anemia, Hb <11 g/dL^3^	64.0	64.0	−0.04 (−4.99, 4.92)	0.988	−0.08 (−5.03, 4.87)	0.975
Number of months receiving EBF^4^	4.96 ± 1.64	5.09 ± 1.38	1.02 (1.00, 1.05)	0.093	1.02 (1.00, 1.05)	0.077
Number of months with wasting^4^	0.23 ± 0.73	0.19 ± 0.62	0.81 (0.60, 1.11)	0.196	0.81 (0.60, 1.11)	0.197

^1^Values are means ± SDs or percentages. At age 6 months, unadjusted and adjusted group differences were estimated by fitting linear regression models for the continuous outcomes^2^, to estimate the mean group difference, and using linear probability models with robust variance estimators for the binary outcomes^3^, to estimate risk difference in percentage points. For the outcomes EBF and wasting episodes during the 6 months follow-up, we fitted Poisson regression models with robust variance estimation to compare study groups by the number of months with the outcome adjusted for log number of months assessed^4^. All models contained allocation to the postnatal intervention, and health center and randomization block as fixed effect to account for clustering by the study design. Adjusted models additionally contained a priori determined set of maternal prognostic factors such as age, parity, gestational age, height, MUAC, BMI, and Hb level at study enrolment.

BEP, balanced energy–protein supplement; CI, confidence interval; EBF, exclusive breastfeeding; Hb, hemoglobin; LAZ, length-for-age z-score; MUAC, mid-upper arm circumference; SD, standard deviation; WAZ, weight-for-age z-score; WLZ, weight-for-length z-score.

In a supplementary analysis using four intervention arms, we found the combined intervention group (BEP/BEP) had a higher LAZ (0.16 SD, 95% CI [0.02 to 0.30], *p* = 0.026) and a lower stunting prevalence at 6 months (−4.05 pp, 95% CI [−7.79 to −0.31], *p* = 0.034), followed by the prenatal only BEP group (BEP/IFA) (LAZ: 0.13 SD, 95% CI [−0.01 to 0.28], *p* = 0.073; stunting: −3.51 pp, 95% CI [−7.50 to 0.49], *p* = 0.085), as compared to the control group for both intervention periods (IFA/IFA) (Table 4). On the other hand, LAZ growth trajectories from birth to 12 months were higher in the groups who received the combined intervention (BEP/BEP) (ES: 0.01 SD/month, 95% CI [0.00 to 0.02], *p* = 0.060) and the postnatal only intervention (IFA/BEP) (ES: 0.01 SD/month, 95% CI [0.00 to 0.02], *p* = 0.055), as compared to the control group for both intervention periods (S2 Fig).

**Table 4:** Four-arm comparison of the effect of BEP supplementation on linear growth at 6 months^1^.

Outcomes	Mean ± SD	Unadjusted difference (95% CI)	*p*	Adjusted difference (95% CI)	*p*
LAZ					
Control (*n =* 426)	−0.62 ± 1.06	Reference		Reference	
Prenatal BEP (*n =* 396)	−0.47 ± 1.03	0.14 (−0.01, 0.29)	0.072	0.13 (−0.01, 0.28)	0.073
Postnatal BEP (*n =* 424)	−0.54 ± 1.07	0.08 (−0.06, 0.23)	0.277	0.07 (−0.07, 0.21)	0.301
Prenatal and postnatal BEP (*n* = 413)	−0.43 ± 1.01	0.19 (0.04, 0.34)	0.012	0.16 (0.02, 0.30)	0.026
Stunting (LAZ <−2 SD), %					
Control (*n* = 426)	9.58	Reference		Reference	
Prenatal BEP (*n* = 396)	5.95	−3.57 (−7.59, 0.44)	0.081	−3.51 (−7.50, 0.49)	0.085
Postnatal BEP (*n* = 424)	8.37	−1.14 (−5.20, 2.91)	0.580	−1.18 (−5.17, 2.80)	0.560
Prenatal and postnatal BEP (*n* = 413)	5.37	−4.22 (−8.01, −0.43)	0.029	−4.05 (−7.79, −0.31)	0.034

^1^Values are means ± SDs or percentages. At age 6 months, unadjusted and adjusted group differences between the control and each of the intervention arms were estimated by fitting linear regression models for the continuous outcomes, and linear probability models with robust variance estimation for the binary outcomes. All models contained health center and randomization block as fixed effect to account for clustering by the study design. Adjusted models additionally contained a priori determined prognostic factors, including maternal age, parity, gestational age, height, MUAC, BMI, and hemoglobin level at study enrolment.

BEP, balanced energy–protein supplement; BMI, body mass index; CI, confidence interval; LAZ, length-for-age z-score; SD, standard deviation.

### Secondary outcomes: Infant growth, nutritional status, and morbidity

**Fig 3:**
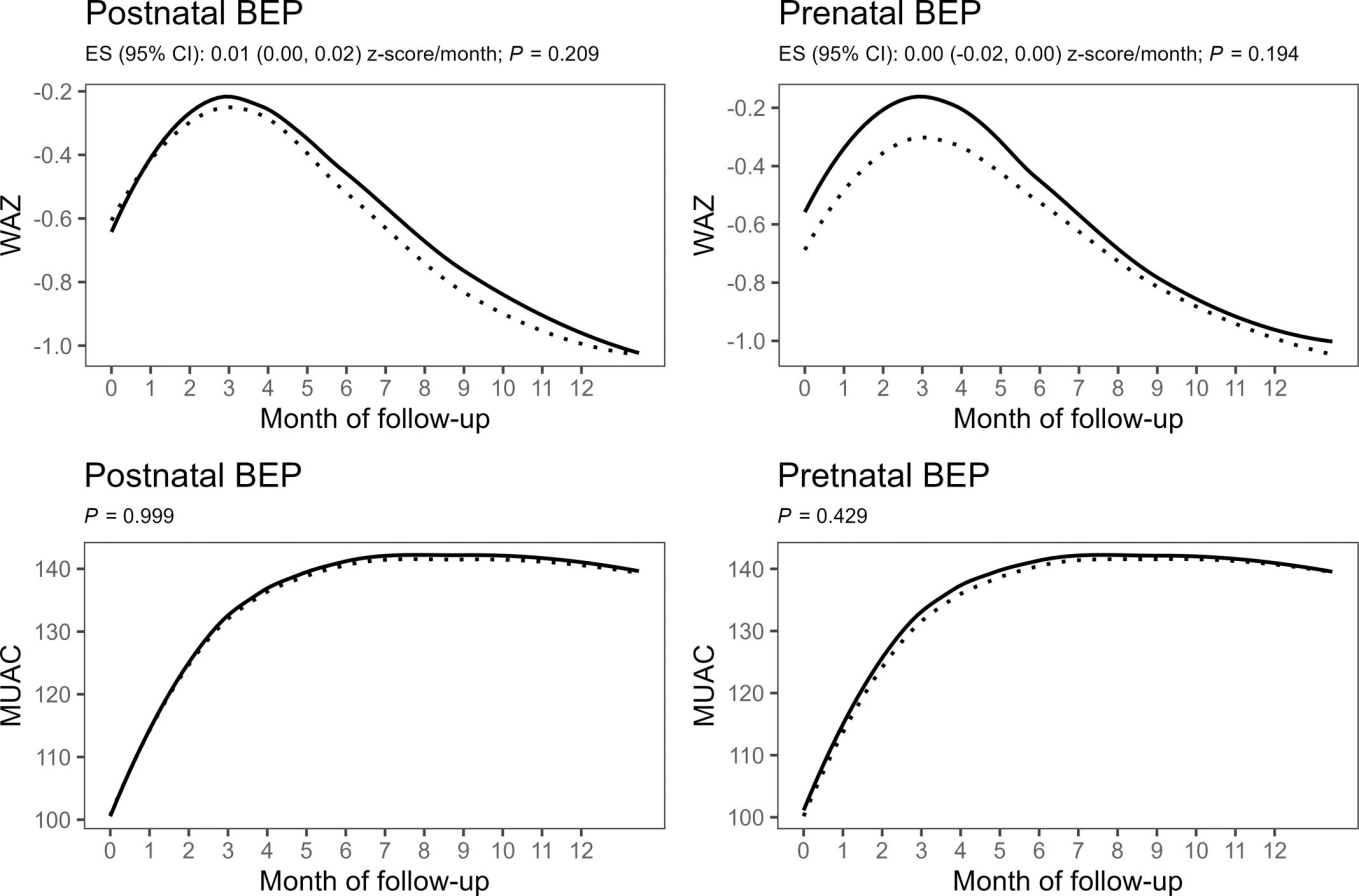
Monthly changes in WAZ (upper panels) and MUAC (lower panels) by postnatal (left panels) and prenatal (right panels) intervention arms with the dashed lines representing the control group (IFA) and solid lines representing the intervention group (IFA + BEP). Line graphs represent locally weighted scatterplot smoothing of observed values. Group differences were estimated using mixed-effects models with random intercepts for infant and random slopes for intervention time, with fixed effects including time (for WAZ), time spline variables with 6 knots (for MUAC), intervention group, and time × group interaction adjusted for allocation to the other intervention, clustering indicators (health center and randomization block), and a priori determined prognostic factors (maternal height, BMI, MUAC, hemoglobin, age and gestational age at inclusion, and parity). For the spline model of MUAC, group difference was tested by likelihood ratio test comparing a model with and without time × group interaction terms. BEP, balanced energy–protein supplement; BMI, body mass index; CI, confidence interval; ES, regression coefficient; IFA, iron–folic acid; MUAC, mid-upper arm circumference; WAZ, weight-for-age Z-score.

**Fig 4:**
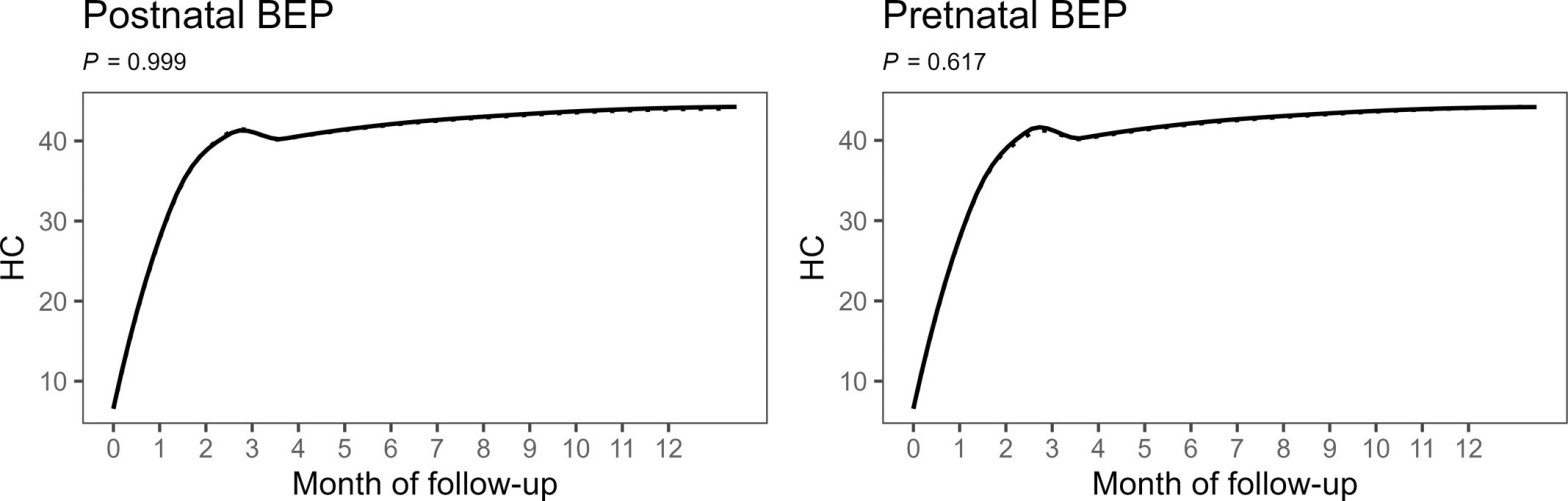
Monthly changes in head circumference by postnatal (left panel) and prenatal (right panel) intervention arms with the dashed lines representing the control group (IFA) and solid lines representing the intervention group (IFA + BEP). Line graphs represent locally weighted scatterplot smoothing of observed values. Group differences were estimated using mixed-effects models with random intercepts for infant and random slopes for intervention time, with fixed effects including time spline variables with 6 knots, intervention group, and time × group interaction terms adjusted for allocation to the other intervention, clustering indicators (health center and randomization block), and a priori determined prognostic factors (maternal height, BMI, MUAC, hemoglobin, age and gestational age at inclusion, and parity). Group difference was tested by likelihood ratio test comparing a model with and without time × group interaction terms. BEP, balanced energy–protein supplement; BMI, body mass index; IFA, iron–folic acid; MUAC, mid-upper arm circumference.

There was no significant effects of the postnatal BEP intervention on the secondary outcomes at 6 months of age, such as WLZ, WAZ, MUAC, head circumference, hemoglobin concentration, and the prevalence rates of wasting, underweight, and anemia (Table 2). Moreover, there were no significant difference between the postnatal intervention and control arms with regard to the number of months infants were exclusively breastfed and the incidence rate of wasting during the 6 months postpartum follow-up. Growth trajectories of WLZ, WAZ, MUAC, and head circumference were also did not differ significantly between the postnatal intervention and control arms (Figs 2–4). Children in the prenatal BEP arm tended towards a lower, but statistically nonsignificant, prevalence of underweight (−2.74 pp, 95% CI [−5.65 to 1.17], *p* = 0.065), whereas no other secondary outcome was affected by the prenatal BEP supplementation (Table 3). The occurrence of common childhood morbidities (i.e., fever, diarrhea, vomit, cough, runny nose, and skin lesions) also did not differ between the control and intervention arms of both the post- and prenatal interventions (Tables 5 and 6).

**Table 5:** Effect of maternal postnatal BEP supplementation on infant morbidity during the 6 months postpartum follow-up^1^.

Outcomes	Control	Intervention	Unadjusted risk ratio (95% CI)	*p*	Adjusted risk ratio (95% CI)	*p*
Fever (measured during visit)						
Child days with event/total child days followed	17/609	18/632				
Longitudinal prevalence (95% CI)	2.77 (2.29, 3.31)	2.78 (2.31, 3.32)	0.98 (0.77, 1.25)	0.881	1.00 (0.78, 1.28)	0.991
Fever (reported by mother)						
Child days with event/total child days followed	39/4255	40/4409				
Longitudinal prevalence (95% CI)	0.40 (0.33, 0.47)	0.40 (0.33, 0.48)	1.02 (0.74, 1.41)	0.906	1.03 (0.75, 1.42)	0.871
Diarrhea						
Child days with event/total child days followed	32/4255	32/4409				
Longitudinal prevalence (95% CI)	5.25 (4.59, 5.99)	5.00 (4.36, 5.70)	1.02 (0.73, 1.43)	0.908	1.04 (0.74, 1.46)	0.817
Vomit						
Child days with event/total child days followed	15/4255	11/4409				
Longitudinal prevalence (95% CI)	2.53 (2.08, 3.06)	1.79 (1.41, 2.23)	0.65 (0.37, 1.15)	0.143	0.76 (0.43, 1.33)	0.340
Cough						
Child days with event/total child days followed	59/4255	52/4409				
Longitudinal prevalence (95% CI)	9.70 (8.80, 10.7)	8.21 (7.39, 9.10)	0.91 (0.71, 1.18)	0.476	0.91 (0.70, 1.17)	0.452
Runny nose						
Child days with event/total child days followed	69/4255	59/4409				
Longitudinal prevalence (95% CI)	11.3 (10.3, 12.3)	9.29 (8.42, 10.2)	0.86 (0.68, 1.09)	0.204	0.85 (0.67, 1.08)	0.173
Skin lesion						
Child days with event/total child days followed	6/4255	6/4409				
Longitudinal prevalence (95% CI)	0.91 (0.65, 1.25)	1.00 (0.72, 1.34)	1.14 (0.48, 2.73)	0.762	1.18 (0.47, 2.98)	0.721

^1^Longitudinal prevalence was calculated using the total number of days that the child presented a positive outcome as the numerator and the total number of days observed or assessed as the denominator. Risk ratios and corresponding *p*-values were estimated using Poisson regression model with robust variance estimation comparing intervention groups by number of days with morbidity adjusted for log number of days observed or assessed. All models were adjusted for allocation to the prenatal BEP supplementation and clustering indicators (health center and randomization block), whereas adjusted models additionally contained a priori determined set of maternal prognostic factors (maternal height, BMI, MUAC, hemoglobin, age and gestational age at inclusion, and parity).

BEP, balanced energy–protein; BMI, body mass index; CI, confidence interval; MUAC, mid-upper arm circumference.

**Table 6:** Effect of maternal prenatal BEP supplementation on infant morbidity during the 6 months postpartum follow-up^1^.

Outcomes	Control	Intervention	Unadjusted risk ratio (95% CI)	*p*	Adjusted risk ratio (95% CI)	*p*
Fever (measured during visits)						
Child weeks with event/total child weeks followed	18/636	17/605				
Longitudinal prevalence (95% CI)	2.78 (2.32, 3.32)	2.76 (2.29, 3.31)	0.94 (0.74, 1.25)	0.637	0.97 (0.75, 1.24)	0.783
Fever (reported by mother)						
Child weeks with event/total child weeks followed	39/4,437	39/4,227				
Longitudinal prevalence (95% CI)	0.40 (0.33, 0.48)	0.40 (0.33, 0.47)	1.09 (0.80, 1.48)	0.574	1.08 (0.80, 1.47)	0.610
Diarrhea						
Child weeks with event/total child weeks followed	29/4,437	35/4,227				
Longitudinal prevalence (95% CI)	4.54 (3.93, 5.21)	5.74 (5.04, 6.51)	1.16 (0.83, 1.62)	0.395	1.17 (0.84, 1.65)	0.351
Vomit						
Child weeks with event/total child weeks followed	12/4,437	15/4,227				
Longitudinal prevalence (95% CI)	1.82 (1.44, 2.26)	2.50 (2.05, 3.03)	1.44 (0.85, 2.43)	0.171	1.47 (0.87, 2.47)	0.147
Cough						
Child weeks with event/total child weeks followed	59/4,437	52/4,227				
Longitudinal prevalence (95% CI)	9.23 (8.36, 10.17)	8.65 (7.78, 9.58)	0.90 (0.70, 1.15)	0.391	0.89 (0.69, 1.14)	0.359
Runny nose						
Child weeks with event/total child weeks followed	69/4,437	58/4,227				
Longitudinal prevalence (95% CI)	10.87 (9.92, 11.9)	9.96 (8.70, 10.6)	0.84 (0.66, 1.07)	0.153	0.83 (0.65, 1.06)	0.144
Skin lesion						
Child weeks with event/total child weeks followed	5/4,437	6/4,227				
Longitudinal prevalence (95% CI)	0.85 (0.60, 1.17)	1.06 (0.78, 1.42)	1.24 (0.53, 2.89)	0.620	1.20 (0.53, 2.74)	0.665

^1^Longitudinal prevalence was calculated using the total number of weeks that the child presented a positive outcome as the numerator and the total number of weeks observed or assessed as the denominator. Risk ratios and corresponding *p*-values were estimated using Poisson regression model with robust variance estimation comparing intervention groups by number of days with morbidity adjusted for log number of days observed or assessed. All models were adjusted for allocation to the postnatal BEP supplementation and clustering indicators (health center and randomization block), whereas adjusted models additionally contained a priori determined set of maternal prognostic factors (maternal height, BMI, MUAC, hemoglobin, age and gestational age at inclusion, and parity).

BEP, balanced energy–protein; BMI, body mass index; CI, confidence interval; MUAC, mid-upper arm circumference.

Finally, our main findings following the intention-to-treat approach were confirmed by both complete cases and per protocol analyses (S4–S7 Tables). The per-protocol analysis did not show stronger effects on study outcomes compared to the main analysis.

## Discussion

The MISAME-III trial indicates that modest increments in size at birth, attained from prenatal fortified BEP supplementation, are sustained at 6 months of age in terms of improved linear growth and lower prevalence of stunting. However, maternal BEP supplementation during the first 6 months postpartum did not result in a significant effect on infant linear growth at the age of 6 months. Nevertheless, we do find an indication for a lagged benefit of postnatal BEP supplementation, as shown by improved monthly growth trajectories, though this was not confirmed by the cross-sectional analysis of the effect at the age of 12 months in a subsample of infants.

The few prior studies that evaluated the impact of maternal LNS supplementation during pregnancy and/or lactation on infant growth found mixed results. Moreover, direct comparisons between our findings on infant growth and those from other LNS trials are difficult, due to heterogeneity in terms of the type and composition of supplements, period and duration of supplementation, and the comparator used as a control group. The most similar RCT, in terms of the type of supplement and study setting, is the MISAME-II trial [37]. In that study, prenatal large-quantity LNS supplementation led to inferior infant growth (i.e., LAZ) during the 12 months postpartum (−0.03 z-score/month, 95% CI [−0.60 to −0.01]), despite newborns in the LNS arm having acquired greater length at birth [16,37]. However, MISAME-II did not include a postnatal supplementation phase and used a more active comparator (MMN tablet, rather than IFA) as a control group. It was hypothesized that a mismatch between better nutritional environment in utero, due to prenatal LNS supplementation, followed by a poorer postnatal nutritional environment might explain the result. Our four-arm analysis also showed a complementary role of the prenatal and early postnatal maternal BEP in preventing growth retardation during the fetal and infant periods. Especially growth trajectories in the combined pre- and postnatal BEP group suggest the complementary effect of the postnatal BEP in preventing a diminishing effect of the prenatal BEP observed towards late infancy, supporting this hypothesis. Furthermore, the MMN tablets used as control supplement likely had a positive and lasting impact on linear growth, masking any effects of the large-quantity LNS [40]. Similarly, a cluster RCT in Niger, which compared prenatal medium-quantity LNS and MMN against IFA, also indicated that prenatal LNS had, with the exception of a small effect on MUAC, no effect on child growth at the age of 24 months (effect on LAZ: 0.04 z-score, 95% CI [−0.22 to 0.30]) [15]. The findings from these and other RCTs suggest that, in the context of low- and middle-income countries (LMICs), prenatal supplementation alone may not be sufficient to prevent an important portion of child growth faltering [41].

Our analysis indicates that LAZ and stunting prevalence at 6 months of age were affected by the prenatal, but not by the postnatal BEP supplementation. Furthermore, there was a modest difference in LAZ growth trajectories between postnatal study arms primarily in late infancy, which, however, did not lead to a significant effect on LAZ at 12 months of age. Other trials evaluated the impact of combined prenatal and postnatal supplementation together with child supplementation. In Ghana [18] and Bangladesh [19], small-quantity LNS provided to women during pregnancy and for 6 months thereafter, followed by supplementation with the same dose of small quantity LNS of their children between the ages of 6 to 24 months was shown to promote child growth at 18 and 24 months, respectively. In contrast, a comparable RCT in Malawi failed to improve child growth by the age of 18 months [20]. Furthermore, the first MISAME trial, also conducted in Houndé, Burkina Faso, showed that MMN supplementation during pregnancy and lactation improved infant growth at 12 months, as compared to IFA tablets (0.13 z-score, 95% CI [0.02 to 0.24]) [40]. The comparable effect sizes on LAZ between MISAME-I and III despite the different intervention supplements (MMN versus fortified BEP) used might suggest the micronutrients compartment in the fortified BEP could be driving the observed improvements in linear growth. On the other hand, it should be noted that there are already 14 years in between the two trials hampering a direct comparison of interventions’ effect. For instance, we observed that anemia prevalence (52% versus 38%) and mean BMI (21 versus 22 kg/m^2^) of the study mothers has improved over these 14 years. The improvements in growth by the postnatal BEP can be understood from the timing of growth faltering during early life. Observational studies have identified the important contributors and the most sensitive periods of growth faltering in LMICs [6,42–44]. Victora and colleagues [6] showed that growth faltering starts in utero and accumulates rapidly until 24 months of age, with the relatively more sensitive periods being fetal growth retardation and growth failure during infancy starting from around 3 months of age. The growth patterns of infants in MISAME-III demonstrate the phenomenon of intrauterine growth restriction, whereas postnatal growth faltering seems to start relatively late (after 6 months) compared to most other settings. The late initiation of growth faltering during infancy might be due to various factors, such as high coverage of EBF as a result of the monthly counselling offered by study midwives, which might have helped in providing the infant with optimal energy and nutrient intakes until 6 months of age and also limiting potential risks of gastrointestinal infections and inflammation arising from early introduction of unhygienic foods [45,46]. Consequently, the benefit of the postnatal BEP supplementation might only have become apparent after 6 months with the introduction of nutritionally suboptimal complementary foods. The lack of strong effect on LAZ at 12 months is likely due to the fact that the intervention period was only limited to the first 6 months postpartum and BEP supplementation consequently not spanning the more vulnerable period for linear growth faltering in the second half of infancy. Hence, findings from MISAME-III suggest that prenatal and early postnatal LNS supplementation alone is insufficient to have long-term impact on child growth faltering.

In addition to the lack of BEP effect on anemia prevalence, it was unexpected that two-third of infants at 6 months were anemic. This is despite the fact that mothers in the control and intervention groups received iron and folic acid in the BEP and IFA tablets during pregnancy and lactation. Moreover, we have previously showed that the prenatal BEP and IFA tablets did not have a preventive effect on the 10 pp increase in maternal anemia observed during pregnancy [47]. Meta-analysis of previous studies also concluded that IFA and MMN were more efficacious at reducing maternal anemia when compared to LNS [41]. The high prevalence of maternal and infant anemia in the presence of maternal BEP and IFA supplementations suggest the need for more in-depth study on the etiologies of anemia and possible dietary factors that might affect nutrient bioavailability in the target population. We did not find any important effect of the pre- or postnatal BEP on the risk of common childhood morbidities. These findings are consistent with results of most previous LNS trials [16,20,48,49]. Only in a few studies selected effects were reported when LNS supplementation was provided to older infants and children [50] and children with increased risk of acute malnutrition [51].

Our subgroup analysis did not show a differential effect of BEP on infant growth by maternal nutritional status using baseline maternal BMI and MUAC measurements. This might be due to the fact that BMI and MUAC measurements mainly reflect calorie intake and are not sensitive to show maternal status of micronutrients including breastmilk nutrient composition and the probably associated infant linear growth and nutritional status. The plasticity of breastmilk composition in the face of maternal undernutrition or nutritional supplementation is as yet not well established [10,52,53]. Hence, future MISAME-III substudies aim to assess the effect of BEP supplements on metabolomic profiles (e.g., nutrients in maternal milk). Moreover, more granular effects of prenatal and postnatal BEP supplementation will be evaluated through body composition analyses of mother–infant dyads (e.g., fat and fat-free mass) and biological markers (e.g., relative telomere lengths), which likely provide additional insights into long-term vulnerability (e.g., premature mortality).

The current study has several strengths that ensure the reliability of the findings. Supplements compliance was closely verified by a community-based network of village workers that home visited the study participants multiple times per week resulting in high adherence to the study supplements. In addition, supplements were taken under observation of these village workers, which limited the risk of sharing with other household members. The high acceptability of the BEP supplement was achieved by implementing a rigorous two-phase formative study [32,33]. Furthermore, dietary intake assessments confirmed that micronutrients requirements were covered by consuming the large-quantity BEP supplement in combination with the maternal basal diet [30]. Moreover, this survey also ruled out any substitution effects of BEP supplement for foods in the usual diet, which might have limited the efficacy of the intervention. Furthermore, breastfeeding and complementary feeding practices were also found to be balanced between intervention arms. The individually randomized 2 × 2 factorial design applied in MISAME-III enabled us to disentangle and separate the efficacy of BEP supplementation during pregnancy and lactation. Lastly, our results following the intention-to-treat principle were robust to both complete cases and per-protocol analyses.

Key limitations of MISAME-III are the nonblinded administration of BEP and IFA supplements and the lack of information on other prognostic factors of infant growth (e.g., maternal infection, asymptomatic inflammation, stress, or physical activity) to determine the extent to which these might have influenced the effect on nutrient availability (e.g., environmental enteric dysfunction) or nutrient sequestration in the mother. In addition, our trial did not collect data on acute or chronic infection in the child, which could have limited the potential benefits on postnatal LAZ. Furthermore, we are unable to assess the efficacy of BEP supplementation on improved child development outcomes [54]. Besides, the MISAME-III study was unable to assess the potential effect of LNS supplementation across the entire window of opportunity (i.e., conception to the second year of life) as BEP supplements were provided only up to 6 months postpartum. Finally, the close study follow-up through daily home visits and other components of the RCT calls for a careful interpretation of the potential of the current intervention under program settings including cost-effectiveness of the study supplements.

In conclusion, MISAME-III provides evidence that linear growth benefits from prenatal BEP supplementation at birth are sustained during early infancy. The maternal BEP supplementation during lactation may also lead to a delayed positive effect on infant linear growth. These findings suggest that BEP supplementation during pregnancy can contribute to the efforts to reduce the high burden of child growth faltering in LMICs.

## Supporting information

S1 CONSORT Checklist CONSORT checklist of the manuscript. (DOC)

S1 Table Nutritional values of the ready-to-use supplementary food for pregnant and lactating women. (DOCX)

S2 Table Breastfeeding and complementary feeding practices by post- and prenatal intervention arms^1^. (DOCX)

S3 Table Effect of prenatal and postnatal BEP supplementation on linear growth in a subsample of infants at 9 and 12 months of follow-up. (DOCX)

S4 Table Effect of maternal postnatal BEP supplementation on infant growth and nutritional status at 6 months (complete cases analysis). (DOCX)

S5 Table Effect of maternal postnatal BEP supplementation on infant growth and nutritional status at 6 months (per-protocol analysis). (DOCX)

S6 Table Effect of maternal prenatal BEP supplementation on infant growth and nutritional status at 6 months (complete cases analysis). (DOCX)

S7 Table Effect of maternal prenatal BEP supplementation on infant growth and nutritional status at 6 months (per-protocol analysis). (DOCX)

S8 Table Subgroup analysis of the efficacy of maternal postnatal BEP supplementation on infant height-for-age z-score at 6 months. (DOCX)

S9 Table Subgroup analysis of the efficacy of maternal prenatal BEP supplementation on infant height-for-age z-score at 6 months. (DOCX)

S1 Fig Trial flowchart of the MISAME-III study by the prenatal intervention arms. BEP, balanced energy–protein supplementation; GA, gestational age; IFA, iron–folic acid tablets; MISAME, MIcronutriments pour la SAnté de la Mère et de l’Enfant.
(DOCX)

S2 Fig Monthly changes in LAZ by four-arm groups including the control group receiving IFA/IFA supplementation (black line), the prenatal only supplementation group receiving BEP/IFA (blue line), the postnatal only supplementation group receiving IFA/BEP (yellow line), and the combined pre- and postnatal supplementation group receiving BEP/BEP (red line). Line graphs represent locally weighted scatterplot smoothing of observed values. Group differences were estimated using mixed-effects models with random intercepts for infant and random slopes for intervention time, with fixed effects including time, quadratic time, intervention group, and time × group interaction adjusted for clustering indicators (health center and randomization block), and a priori determined prognostic factors (maternal height, BMI, MUAC, hemoglobin, age and gestational age at inclusion, and parity). Compared to the control group, effect sizes in the prenatal only supplementation group (ES: 0.005 SD/month, 95% CI: −0.007 to 0.017, *p* = 0.416), the postnatal only supplementation group (ES: 0.012 SD/month, 95% CI: 0.000 to 0.024, *p* = 0.055), and the combined pre- and postnatal supplementation group (ES: 0.011 SD/month, 95% CI: 0.000 to 0.023, *p* = 0.060). BEP, balanced energy–protein supplement; BMI, body mass index; CI, confidence interval; ES, effect size (regression coefficient); IFA, iron–folic acid; LAZ, length-for-age Z-score; MUAC, mid-upper arm circumference; SD, standard deviation.
(DOCX)

S1 Supporting Information Inclusivity in global research. (DOCX)

S1 Statistical Analysis plan Statistical analysis plan: Impact of a prenatal and postnatal balanced energy–protein supplement on birth size and postnatal child growth in Burkina Faso. (PDF)

## Abbreviations

BEP: balanced energy–protein
BMI: body mass index
CI: confidence interval
EARs: Estimated Average Requirements
EBF: exclusive breastfeeding
IFA: iron–folic acidsss
LAZ: length-for-age z-score
LMICs: low- and middle-income countries
LNS: lipid-based nutrient supplement
MISAME: MIcronutriments pour la SAnté de la Mère et de l’Enfant
mITT: modified intention-to-treat
MMN: multimicronutrient tablet
MUAC: mid-upper arm circumference
pp: percentage points
RCT: randomized controlled trial
SD: standard deviation
WAZ: weight-for-age z-score
WHO: World Health Organization
WLZ: weight-for-length z-score
