# Correction: Fortified balanced energy–protein supplementation during pregnancy and lactation and infant growth in rural Burkina Faso: A 2 × 2 factorial individually randomized controlled trial

**DOI:** 10.1371/journal.pmed.1004267

**Published:** 2023-07-17

**Authors:** Alemayehu Argaw, Brenda de Kok, Laeticia Celine Toe, Giles Hanley-Cook, Trenton Dailey-Chwalibóg, Moctar Ouédraogo, Anderson Compaoré, Katrien Vanslambrouck, Rasmané Ganaba, Patrick Kolsteren, Carl Lachat, Lieven Huybregts

In the sub-section Intervention and procedures, within the Methods section, the fifth sentence contains an error. The correct sentence is as follows: A daily dose of the BEP (72 g) provided an energy top up of 393 kcal and consisted of 36% lipids, 20% protein, and 32% carbohydrates [34]. The supplement covered at least the EARs of pregnant women for 11 micronutrients, except calcium, phosphorous, and magnesium.
