# Supplementary material for: Fortified balanced energy–protein supplementation during pregnancy and lactation and infant growth in rural Burkina Faso: A 2 × 2 factorial individually randomized controlled trial
Source: PLoS Med. 2023 Feb 6;20(2):e1004186. doi: 10.1371/journal.pmed.1004186 (PMC9943012; doi:10.1371/journal.pmed.1004186)
Supplement: S1 Table — (DOCX) [file pmed.1004186.s002.docx]

**Table S1. Nutritional values of the ready-to-use supplementary food for pregnant and lactating women^1^**

|  | **Mean for 72g (serving size)** |
| --- | --- |
| Total energy (kcal) | 393 |
| Lipids (g) | 26 |
| Linoleic acid (g) | 3.9 |
| α-Linoleic acid (g) | 1.3 |
| Proteins (g) | 14.5 |
| Carbohydrates (g) | 23.3 |
| Calcium (mg) | 500 |
| Copper (mg) | 1.3 |
| Phosphorus (mg) | 418 |
| Iodine (µg) | 250 |
| Iron (mg) | 22 |
| Selenium (µg) | 65 |
| Manganese (mg) | 2.1 |
| Magnesium (mg) | 73 |
| Potassium (mg) | 562 |
| Zinc (mg) | 15 |
| Vitamin A (µg RE)^2^ | 770 |
| Thiamin (mg) | 1.4 |
| Riboflavin (mg) | 1.4 |
| Niacin (mg) | 15 |
| Vitamin B5 (mg) | 7 |
| Vitamin B6 (mg) | 1.9 |
| Folic acid (µg) | 400 |
| Vitamin B12 (mg) | 2.6 |
| Vitamin C (mg) | 100 |
| Vitamin D (µg cholecalciferol)^3^ | 15 |
| Vitamin E (mg α-tocopherol)^4^ | 18 |
| Vitamin K (µg) | 72 |

^1^Ingredients: vegetable oils (rapeseed, palm, soy in varying proportions), defatted soy flour, skimmed milk powder, peanuts, sugar, maltodextrin, soy protein isolate, vitamin and mineral complex, stabilizer (fully hydrogenated vegetable fat, mono and diglycerides).

^2^1 µg vitamin A RE = 3.333 IU vitamin A.

^3^1 μg cholecalciferol = 40 IU vitamin D.

^4^1 mg α-tocopherol = 2,22 IU vitamin E.

IU, international unit; RE, retinol equivalen
