# Supplementary material for: Fortified balanced energy–protein supplementation during pregnancy and lactation and infant growth in rural Burkina Faso: A 2 × 2 factorial individually randomized controlled trial
Source: PLoS Med. 2023 Feb 6;20(2):e1004186. doi: 10.1371/journal.pmed.1004186 (PMC9943012; doi:10.1371/journal.pmed.1004186)
Supplement: S2 Table — (DOCX) [file pmed.1004186.s003.docx]

**Table S2. Breastfeeding and complementary feeding practices by post- and prenatal intervention arms^1^**

| **Characteristics** | **Postnatal intervention** | | ***P*^3^** |  | **Prenatal intervention** | | ***P*^3^** |
| --- | --- | --- | --- | --- | --- | --- | --- |
|  | **Control (*n* = 822)** | **Intervention (*n* = 837)** |  |  | **Control (*n* = 850)** | **Intervention (*n* = 809)** |  |
| Child ever breastfed | 96.6 | 97.3 | 0.437 |  | 97.1 | 96.8 | 0.711 |
| DDS at 9 mo, 1-8 food groups^2^ | 2.53 ± 1.23 | 2.60 ± 1.27 | 0.150 |  | 2.51 ± 1.22 | 2.63 ± 1.28 | 0.213 |
| DDS at 12 mo, 1-8 food groups^2^ | 2.97 ± 1.27 | 3.06 ± 1.31 | 0.114 |  | 2.95 ± 1.26 | 3.08 ± 1.32 | 0.238 |
| Minimum DDS at 9 mo, ≥ 5 food groups^2^ | 6.08 | 7.28 | 0.356 |  | 5.97 | 7.43 | 0.349 |
| Minimum DDS at 12 mo, ≥ 5 food groups^2^ | 9.05 | 12.08 | 0.072 |  | 9.78 | 11.45 | 0.448 |
| Egg and/or flesh food consumption at 9 mo^2^ | 23.3 | 25.9 | 0.262 |  | 23.6 | 25.7 | 0.379 |
| Egg and/or flesh food consumption at 12 mo^2^ | 36.6 | 41.6 | 0.121 |  | 38.2 | 40.1 | 0.585 |
| No vegetable or fruit consumption at 9 mo^2^ | 46.2 | 44.9 | 0.344 |  | 46.7 | 44.5 | 0.566 |
| No vegetable or fruit consumption at 12 mo^2^ | 19.6 | 16.9 | 0.376 |  | 20.1 | 16.2 | 0.139 |

^1^Data are percentages or means ± SD.

^2^Data are based on a subsample of participants at 9 (n = 1333) and 12 months (n = 955). Assessed using the WHO IYCF indicators (WHO & UNICEF, 2021).

^3^P-values comparing intervention and control groups were based on linear regression models for the continuous outcomes and linear probability modes with robust variance estimation for the binary outcome. All models are adjusted for health center and randomization block.

DDS, dietary diversity score

.
