# Supplementary material for: Fortified balanced energy–protein supplementation during pregnancy and lactation and infant growth in rural Burkina Faso: A 2 × 2 factorial individually randomized controlled trial
Source: PLoS Med. 2023 Feb 6;20(2):e1004186. doi: 10.1371/journal.pmed.1004186 (PMC9943012; doi:10.1371/journal.pmed.1004186)
Supplement: S3 Table — (DOCX) [file pmed.1004186.s004.docx]

**Table S3. Effect of prenatal and postnatal BEP supplementation on linear growth in a subsample of infants at 9 and 12 months of follow-up^1^**

| **Outcomes** | **Control** | **Intervention** | **Unadjusted difference (95% CI)** | ***p*** | **Adjusted difference (95% CI)** | ***p*** |
| --- | --- | --- | --- | --- | --- | --- |
| **At 9 months follow-up** |  |  |  |  |  |  |
| Postnatal BEP (*n*) | **679** | **663** |  |  |  |  |
| Length-for-age z-score (LAZ) | -0.62 ± 1.04 | -0.56 ± 1.05 | 0.06 (-0.05, 0.18) | 0.273 | 0.04 (-0.07, 0.14) | 0.517 |
| Stunting (LAZ < -2 SD), % | 8.25 | 7.99 | 0.14 (-3.06, 2.77) | 0.924 | 0.17 (-2.72, 3.07) | 0.907 |
| Prenatal BEP (*n*) | **692** | **650** |  |  |  |  |
| Length-for-age z-score (LAZ) | -0.64 ± 1.07 | -0.54 ± 1.01 | 0.10 (-0.01, 0.22) | 0.071 | 0.08 (-0.03, 0.18) | 0.153 |
| Stunting (LAZ < -2 SD), % | 9.68 | 6.46 | -3.28 (-6.23, -0.32) | 0.030 | -2.91 (-5.85, 0.02) | 0.052 |
| **At 12 months follow-up** |  |  |  |  |  |  |
| Postnatal BEP (n) | **475** | **479** |  |  |  |  |
| Length-for-age z-score (LAZ) | -0.90 ± 1.02 | -0.81 ± 0.97 | 0.10 (-0.03, 0.22) | 0.129 | 0.06 (-0.06, 0.18) | 0.330 |
| Stunting (LAZ < -2 SD), % | 11.0 | 10.0 | -1.53 (-5.40, 2.35) | 0.439 | -0.67 (-4.52, 3.16) | 0.731 |
| Prenatal BEP (n) | **501** | **453** |  |  |  |  |
| Length-for-age z-score (LAZ) | -0.88 ± 1.03 | -0.83 ± 0.97 | 0.05 (-0.07, 0.18) | 0.408 | 0.02 (-0.10, 0.14) | 0.699 |
| Stunting (LAZ < -2 SD), % | 11.8 | 9.05 | -3.09 (-7.02, 0.83) | 0.122 | -2.39 (-6.22, 1.43) | 0.220 |

^1^Values are means ± SDs or percentages. In a subsample of infants at the age of 9 and 12 months, unadjusted and adjusted group differences were estimated by fitting linear regression model (length-for-age) and linear probability model with robust variance estimation (stunting). All models contained health center and randomization block as fixed effect to account for clustering by the study design. Adjusted models additionally contained *a priori* determined set of maternal prognostic factors such as age, parity, gestational age, height, mid-upper arm circumference, body mass index, and hemoglobin level at study enrolment. BEP, balanced protein-energy supplement.
