# Supplementary material for: Fortified balanced energy–protein supplementation during pregnancy and lactation and infant growth in rural Burkina Faso: A 2 × 2 factorial individually randomized controlled trial
Source: PLoS Med. 2023 Feb 6;20(2):e1004186. doi: 10.1371/journal.pmed.1004186 (PMC9943012; doi:10.1371/journal.pmed.1004186)
Supplement: S4 Table — (DOCX) [file pmed.1004186.s005.docx]

**Table S4. Effect of maternal postnatal BEP supplementation on infant growth and nutritional status at 6 months (complete cases analysis)^1^**

| **Outcomes** | **Control (*n* = 716)** | **Intervention (*n* = 746)** | **Unadjusted difference (95% CI)** | ***p*** | **Adjusted difference (95% CI)** | ***p*** |
| --- | --- | --- | --- | --- | --- | --- |
| Length-for-age z-score (LAZ)^2^ | -0.50 ± 1.04 | -0.44 ± 1.03 | 0.06 (-0.05, 0.16) | 0.285 | 0.04 (-0.06, 0.14) | 0.390 |
| Weight-for-length z-score (WLZ)^2^ | -0.28 ± 1.14 | -0.21 ± 1.16 | 0.08 (-0.04, 0.19) | 0.199 | 0.07 (-0.04, 0.19) | 0.225 |
| weight-for-age z-score (WAZ)^2^ | -0.57 ± 1.12 | -0.49 ± 1.10 | 0.10 (-0.02, 0.21) | 0.095 | 0.08 (-0.03, 0.20) | 0.133 |
| Arm circumference, mm^2^ | 140 ± 11.8 | 141 ± 12.1 | 0.82 (-0.36, 2.01) | 0.175 | 0.84 (-0.32, 2.00) | 0.157 |
| Head circumference, cm^2^ | 42.0 ± 1.42 | 42.1 ± 1.48 | 0.08 (-0.06, 0.23) | 0.269 | 0.07 (-0.07, 0.22) | 0.312 |
| Hemoglobin (Hb), g/dL^2^ | 10.5 ± 1.37 | 10.4 ± 1.34 | -0.08 (-0.22, 0.05) | 0.236 | -0.09 (-0.23, 0.05) | 0.214 |
| Stunting (LAZ < -2 SD), %^3^ | 7.12 | 6.03 | -1.02 (-3.59, 1.56) | 0.438 | -0.98 (-3.51, 1.54) | 0.444 |
| Wasting (WLZ < -2 SD), %^3^ | 6.01 | 6.17 | 0.11 (2.33, 2.54) | 0.931 | 0.28 (-2.16, 2.71) | 0.823 |
| Underweight (WAZ < -2 SD), %^3^ | 8.94 | 7.79 | -1.39 (-4.21, 1.42) | 0.332 | -1.14 (-3.93, 1.65) | 0.423 |
| Anemia, Hb < 11 g/dL^3^ | 62.2 | 65.7 | 3.18 (-1.90, 8.27) | 0.220 | 3.19 (-1.91, 8.28) | 0.220 |
| Number of months receiving EBF^4^ | 4.64 ± 1.57 | 4.74 ± 1.53 | 1.01 (0.98, 1.03) | 0.650 | 1.01 (0.98, 1.03) | 0.596 |
| Number of months with wasting^4^ | 0.17 ± 0.67 | 0.17 ± 0.63 | 0.97 (0.68, 1.41) | 0.891 | 0.96 (0.66, 1.38) | 0.809 |

^1^Values are means ± SDs or percentages. At age six months, unadjusted and adjusted group differences were estimated by fitting linear regression models for the continuous outcomes^2^, to estimate the mean group difference, and using linear probability models with robust variance estimators for the binary outcomes^3^, to estimate risk difference in percentage points. For the outcomes exclusive breastfeeding and wasting episodes during the six months follow-up, we fitted Poisson regression models with robust variance estimation to compare study groups by the number of months with the outcome adjusted for log number of months assessed^4^. All models contained allocation to the prenatal intervention, and health center and randomization block as fixed effect to account for clustering by the study design. Adjusted models additionally contained *a priori* determined set of maternal prognostic factors such as age, parity, gestational age, height, mid-upper arm circumference, body mass index and hemoglobin level at study enrolment. BEP, balanced protein-energy supplement; CI, confidence interval; EBF, exclusive breastfeeding; SD, standard deviation.
