# Supplementary material for: Fortified balanced energy–protein supplementation during pregnancy and lactation and infant growth in rural Burkina Faso: A 2 × 2 factorial individually randomized controlled trial
Source: PLoS Med. 2023 Feb 6;20(2):e1004186. doi: 10.1371/journal.pmed.1004186 (PMC9943012; doi:10.1371/journal.pmed.1004186)
Supplement: S5 Table — (DOCX) [file pmed.1004186.s006.docx]

**Table S5. Effect of maternal postnatal BEP supplementation on infant growth and nutritional status at 6 months (per-protocol analysis)^1^**

| **Outcomes** | **Control (*n* = 716)** | **Intervention (*n* = 607)** | **Unadjusted difference (95% CI)** | ***p*** | **Adjusted difference (95% CI)** | ***p*** |
| --- | --- | --- | --- | --- | --- | --- |
| Length-for-age z-score (LAZ)^2^ | -0.50 ± 1.04 | -0.46 ± 1.07 | 0.05 (-0.07, 0.16) | 0.427 | 0.04 (-0.07, 0.14) | 0.526 |
| Weight-for-length z-score (WLZ)^2^ | -0.28 ± 1.14 | -0.24 ± 1.18 | 0.05 (-0.07, 0.18) | 0.415 | 0.05 (-0.07, 0.17) | 0.421 |
| weight-for-age z-score (WAZ)^2^ | -0.57 ± 1.12 | -0.52 ± 1.12 | 0.07 (-0.05, 0.19) | 0.267 | 0.06 (-0.06, 0.18) | 0.312 |
| Arm circumference, mm^2^ | 140 ± 11.8 | 140 ± 12.2 | 0.49 (-0.77, 1.75) | 0.445 | 0.54 (-0.70, 1.88) | 0.391 |
| Head circumference, cm^2^ | 42.0 ± 1.42 | 42.0 ±1.51 | 0.05 (-0.11, 0.20) | 0.561 | 0.04 (-0.11, 0.20) | 0.568 |
| Hemoglobin (Hb), g/dL^2^ | 10.5 ± 1.37 | 10.3 ± 1.33 | -0.06 (-0.21, 0.09) | 0.421 | -0.06 (-0.21, 0.08) | 0.397 |
| Stunting (LAZ < -2 SD), %^3^ | 7.12 | 6.43 | -0.80 (-3.57, 1.97) | 0.570 | -0.80 (-3.52, 1.92) | 0.564 |
| Wasting (WLZ < -2 SD), %^3^ | 6.01 | 6.60 | 0.49 (-2.14, 3.11) | 0.717 | 0.58 (-2.05, 3.21) | 0.666 |
| Underweight (WAZ < -2 SD), %^3^ | 8.94 | 8.58 | -0.78 (-3.80, 2.25) | 0.614 | -0.63 (-3.63, 2.37) | 0.681 |
| Anemia, Hb < 11 g/dL^3^ | 62.2 | 66.2 | 2.12 (-3.27, 7.50) | 0.440 | 2.12 (-3.28, 7.52) | 0.441 |
| Number of months receiving EBF^4^ | 4.64 ± 1.57 | 4.77 ± 1.59 | 1.00 (0.97, 1.03) | 0.952 | 1.00 (0.97, 1.03) | 0.950 |
| Number of months with wasting^4^ | 0.17 ± 0.67 | 0.18 ± 0.63 | 0.96 (0.65, 1.41) | 0.840 | 0.92 (0.63, 1.35) | 0.678 |

^1^Values are means ± SDs or percentages. At age six months, unadjusted and adjusted group differences were estimated by fitting linear regression models for the continuous outcomes^2^, to estimate the mean group difference, and using linear probability models with robust variance estimators for the binary outcomes^3^, to estimate risk difference in percentage points. For the outcomes exclusive breastfeeding and wasting episodes during the six months follow-up, we fitted Poisson regression models with robust variance estimation to compare study groups by the number of months with the outcome adjusted for log number of months assessed^4^. All models contained allocation to the prenatal intervention, and health center and randomization block as fixed effect to account for clustering by the study design. Adjusted models additionally contained *a priori* determined set of maternal prognostic factors such as age, parity, gestational age, height, mid-upper arm circumference, body mass index and hemoglobin level at study enrolment. BEP, balanced protein-energy supplement; CI, confidence interval; EBF, exclusive breastfeeding; SD, standard deviation.
