# Supplementary material for: Fortified balanced energy–protein supplementation during pregnancy and lactation and infant growth in rural Burkina Faso: A 2 × 2 factorial individually randomized controlled trial
Source: PLoS Med. 2023 Feb 6;20(2):e1004186. doi: 10.1371/journal.pmed.1004186 (PMC9943012; doi:10.1371/journal.pmed.1004186)
Supplement: S6 Table — (DOCX) [file pmed.1004186.s007.docx]

**Table S6. Effect of maternal prenatal BEP supplementation on infant growth and nutritional status at 6 months (complete cases analysis)^1^**

| **Outcomes** | **Control (*n* = 753)** | **Intervention (*n* = 709)** | **Unadjusted difference (95% CI)** | ***p*** | **Adjusted difference (95% CI)** | ***p*** |
| --- | --- | --- | --- | --- | --- | --- |
| Length-for-age z-score (LAZ)^2^ | -0.53 ± 1.06 | -0.40 ± 1.01 | 0.12 (0.02, 0.23) | 0.023 | 0.10 (0.00, 0.20) | 0.048 |
| Weight-for-length z-score (WLZ)^2^ | -0.24 ± 1.15 | -0.25 ± 1.15 | 0.00 (-0.12, 0.11) | 0.965 | -0.01 (-0.13, 0.10) | 0.836 |
| Weight-for-age z-score (WAZ)^2^ | -0.56 ± 1.14 | -0.50 ± 1.09 | 0.07 (-0.04, 0.19) | 0.202 | 0.05 (-0.06, 0.16) | 0.330 |
| Arm circumference, mm^2^ | 140 ± 12.1 | 141 ± 11.8 | 1.04 (-0.14, 2.22) | 0.084 | 0.90 (-0.26, 2.07) | 0.127 |
| Head circumference, cm^2^ | 42.0 ± 1.53 | 42.1 ± 1.35 | 0.14 (-0.01, 0.28) | 0.069 | 0.12 (-0.02, 0.27) | 0.091 |
| Hemoglobin (Hb), g/dL^2^ | 10.4 ± 1.36 | 10.4 ± 1.35 | 0.01 (-0.13, 0.14) | 0.925 | 0.01 (-0.13, 0.14) | 0.925 |
| Stunting (LAZ < -2 SD), %^3^ | 8.23 | 4.80 | -3.34 (-5.86, -0.81) | 0.010 | -3.14 (-5.62, -0.66) | 0.013 |
| Wasting (WLZ < -2 SD), %^3^ | 6.12 | 6.06 | -0.19 (-2.65, 2.26) | 0.877 | 0.00 (-2.46, 2.46) | 0.999 |
| Underweight (WAZ < -2 SD), %^3^ | 9.57 | 7.05 | -2.86 (-5.71, -0.02) | 0.048 | -2.48 (-5.28, 0.33) | 0.083 |
| Anemia, Hb < 11 g/dL^3^ | 64.0 | 64.1 | 0.32 (-4.73, 5.36) | 0.902 | 0.23 (-4.82, 5.29) | 0.928 |
| Number of months receiving EBF^4^ | 4.63 ± 1.65 | 4.76 ± 1.44 | 1.03 (1.00, 1.05) | 0.054 | 1.03 (1.00, 1.05) | 0.044 |
| Number of months with wasting^4^ | 0.18 ± 0.69 | 0.16 ± 0.60 | 0.86 (0.60, 1.23) | 0.407 | 0.87 (0.61, 1.24) | 0.443 |

^1^Values are means ± SDs or percentages. At age six months, unadjusted and adjusted group differences were estimated by fitting linear regression models for the continuous outcomes^2^, to estimate the mean group difference, and using linear probability models with robust variance estimators for the binary outcomes^3^, to estimate risk difference in percentage points. For the outcomes exclusive breastfeeding and wasting episodes during the six months follow-up, we fitted Poisson regression models with robust variance estimation to compare study groups by the number of months with the outcome adjusted for log number of months assessed^4^. All models contained allocation to the postnatal intervention, and health center and randomization block as fixed effect to account for clustering by the study design. Adjusted models additionally contained *a priori* determined set of maternal prognostic factors such as age, parity, gestational age, height, mid-upper arm circumference, body mass index and hemoglobin level at study enrolment. BEP, balanced protein-energy supplement; CI, confidence interval; EBF, exclusive breastfeeding; SD, standard deviation.
