# Supplementary material for: Fortified balanced energy–protein supplementation during pregnancy and lactation and infant growth in rural Burkina Faso: A 2 × 2 factorial individually randomized controlled trial
Source: PLoS Med. 2023 Feb 6;20(2):e1004186. doi: 10.1371/journal.pmed.1004186 (PMC9943012; doi:10.1371/journal.pmed.1004186)
Supplement: S7 Table — (DOCX) [file pmed.1004186.s008.docx]

**Table S7. Effect of maternal prenatal BEP supplementation on infant growth and nutritional status at 6 months (per-protocol analysis)^1^**

| **Outcomes** | **Control (*n* = 753)** | **Intervention (*n* = 556)** | **Unadjusted difference (95% CI)** | ***p*** | **Adjusted difference (95% CI)** | ***p*** |
| --- | --- | --- | --- | --- | --- | --- |
| Length-for-age z-score (LAZ)^2^ | -0.53 ± 1.06 | -0.41 ± 1.02 | 0.12 (0.01, 0.24) | 0.035 | 0.11 (0.00, 0.22) | 0.054 |
| Weight-for-length z-score (WLZ)^2^ | -0.24 ± 1.15 | -0.26 ± 1.16 | -0.01 (-0.14, 0.12) | 0.844 | -0.02 (-0.14, 0.11) | 0.762 |
| weight-for-age z-score (WAZ)^2^ | -0.56 ± 1.14 | -0.51 ± 1.09 | 0.07 (-0.06, 0.19) | 0.293 | 0.05 (-0.07, 0.17) | 0.392 |
| Arm circumference, mm^2^ | 140 ± 12.1 | 140 ± 11.8 | 1.04 (-0.24, 2.32) | 0.112 | 0.91 (-0.35, 2.17) | 0.156 |
| Head circumference, cm^2^ | 42.0 ± 1.53 | 42.1 ±1.37 | 0.14 (-0.02, 0.30) | 0.087 | 0.13 (-0.02, 0.29) | 0.098 |
| Hemoglobin (Hb), g/dL^2^ | 10.4 ± 1.36 | 10.3 ± 1.31 | -0.03 (-0.18, 0.12) | 0.711 | -0.02 (-0.17, 0.12) | 0.741 |
| Stunting (LAZ < -2 SD), %^3^ | 8.23 | 4.68 | -3.72 (-6.37, -1.06) | 0.006 | -3.55 (-6.17, -0.93) | 0.008 |
| Wasting (WLZ < -2 SD), %^3^ | 6.12 | 5.94 | -0.54 (-3.13, 2.04) | 0.680 | -0.44 (-3.02, 2.15) | 0.740 |
| Underweight (WAZ < -2 SD), %^3^ | 9.57 | 7.55 | -2.61 (-5.69, 0.47) | 0.096 | -2.39 (-5.43, 0.65) | 0.124 |
| Anemia, Hb < 11 g/dL^3^ | 64.0 | 67.1 | 1.87 (-3.55, 7.29) | 0.498 | 1.78 (-3.65, 7.22) | 0.520 |
| Number of months receiving EBF^4^ | 4.63 ± 1.65 | 4.78 ± 1.49 | 1.02 (1.00, 1.05) | 0.105 | 1.02 (1.00, 1.05) | 0.090 |
| Number of months with wasting^4^ | 0.18 ± 0.69 | 0.17 ± 0.63 | 0.83 (0.57, 1.23) | 0.354 | 0.85 (0.58, 1.26) | 0.425 |

^1^Values are means ± SDs or percentages. At age six months, unadjusted and adjusted group differences were estimated by fitting linear regression models for the continuous outcomes^2^, to estimate the mean group difference, and using linear probability models with robust variance estimators for the binary outcomes3, to estimate risk difference in percentage points. For the outcomes exclusive breastfeeding and wasting episodes during the six months follow-up, we fitted Poisson regression models with robust variance estimation to compare study groups by the number of months with the outcome adjusted for log number of months assessed^4^. All models contained allocation to the postnatal intervention, and health center and randomization block as fixed effect to account for clustering by the study design. Adjusted models additionally contained *a priori* determined set of maternal prognostic factors such as age, parity, gestational age, height, mid-upper arm circumference, body mass index and hemoglobin level at study enrolment. BEP, balanced protein-energy supplement; CI, confidence interval; EBF, exclusive breastfeeding; SD, standard deviation.
