# Supplementary material for: Fortified balanced energy–protein supplementation during pregnancy and lactation and infant growth in rural Burkina Faso: A 2 × 2 factorial individually randomized controlled trial
Source: PLoS Med. 2023 Feb 6;20(2):e1004186. doi: 10.1371/journal.pmed.1004186 (PMC9943012; doi:10.1371/journal.pmed.1004186)
Supplement: S8 Table — (DOCX) [file pmed.1004186.s009.docx]

**Table S8. Subgroup analysis of the efficacy of maternal postnatal BEP supplementation on infant length-for-age z score at 6 months^1^**

| **Subgroup factor** | **Control** | |  | **Intervention** | | **Unadjusted *p*** | **Adjusted *p*** |
| --- | --- | --- | --- | --- | --- | --- | --- |
|  | ***n*** | **Mean ± SD** |  | ***n*** | **Mean ± SD** |  |  |
| Maternal age |  |  |  |  |  | 0.168 | 0.178 |
| <20 years | 139 | -0.47 ± 1.06 |  | 167 | -0.37 ± 1.00 |  |  |
| ≥20 years | 577 | -0.60 ± 0.97 |  | 579 | -0.66 ± 1.12 |  |  |
| Child sex |  |  |  |  |  | 0.584 | 0.712 |
| Male | 333 | -0.57 ± 1.04 |  | 376 | -0.50 ± 1.08 |  |  |
| Female | 383 | -0.43 ± 1.04 |  | 370 | -0.38 ± 0.98 |  |  |
| Maternal hemoglobin level |  |  |  |  |  | 0.255 | 0.584 |
| ≥11 g/dL | 448 | -0.44 ± 1.05 |  | 487 | -0.43 ± 1.03 |  |  |
| <11 g/dL (anemic) | 268 | -0.59 ± 1.03 |  | 259 | -0.46 ± 1.03 |  |  |
| Maternal BMI |  |  |  |  |  | 0.781 | 0.386 |
| ≥18.5 kg/m^2^ | 666 | -0.48 ± 1.05 |  | 698 | -0.43 ± 1.04 |  |  |
| <18.5 kg/m^2^ (underweight) | 49 | -0.65 ± 0.97 |  | 48 | -0.60 ± 0.89 |  |  |
| Maternal MUAC |  |  |  |  |  | 0.994 | 0.845 |
| ≥23 cm | 333 | -0.38 ± 1.07 |  | 315 | -0.31 ± 1.00 |  |  |
| <23 cm | 383 | -0.60 ± 1.01 |  | 431 | -0.54 ±1.04 |  |  |
| Maternal height |  |  |  |  |  | 0.622 | 0.849 |
| ≥155 cm | 642 | -0.42 ± 1.03 |  | 684 | -0.39 ± 1.01 |  |  |
| <155 cm | 74 | -1.15 ± 0.95 |  | 62 | -1.02 ± 1.08 |  |  |
| Primiparity |  |  |  |  |  | 0.316 | 0.216 |
| No | 579 | -0.46 ± 1.05 |  | 581 | -0.37 ± 0.97 |  |  |
| Yes | 137 | -0.64 ± 1.00 |  | 165 | -0.69 ± 1.19 |  |  |
| Maternal depression possible |  |  |  |  |  | 0.475 | 0.320 |
| No | 701 | -0.49 ± 1.04 |  | 733 | -0.44 ± 1.03 |  |  |
| Yes | 15 | -0.78 ± 1.27 |  | 13 | -0.40 ± 1.30 |  |  |
| Maternal depression probable |  |  |  |  |  | 0.339 | 0.577 |
| No | 658 | -0.48 ± 1.04 |  | 693 | -0.44 ± 1.04 |  |  |
| Yes | 58 | -0.72 ± 1.02 |  | 53 | -0.46 ± 0.89 |  |  |
| Inter-pregnancy interval |  |  |  |  |  | 0.774 | 0.582 |
| ≥18 months | 698 | -0.50 ± 1.05 |  | 726 | -0.44 ± 1.04 |  |  |
| <18 months | 18 | -0.41 ± 0.88 |  | 20 | -0.23 ± 0.85 |  |  |
| Season of delivery |  |  |  |  |  | 0.799 | 0.312 |
| Plenty | 485 | -0.51 ± 1.07 |  | 509 | -0.44 ± 1.03 |  |  |
| Lean (June – September) | 231 | -0.48 ± 0.99 |  | 237 | -0.44 ± 1.04 |  |  |
| Household food security |  |  |  |  |  | 0.483 | 0.473 |
| Food secure | 319 | -0.45 ± 1.03 |  | 335 | -0.41 ± 1.01 |  |  |
| Food insecure | 397 | -0.53 ± 1.05 |  | 411 | -0.46 ± 1.05 |  |  |

Linear regression models were fitted to test interaction between postnatal BEP supplementation group and a subgroup factor at *P*<0.10. All models contained allocation to the prenatal BEP supplementation, and health center and randomization block as fixed effect to account for clustering by the study design. Adjusted models additionally contained *a priori* determined set of maternal prognostic factors such as age, parity, gestational age, height, mid-upper arm circumference, body mass index and hemoglobin level at study enrolment. BEP, Balanced Energy-Protein Supplementation; BMI, body mass index; MUAC, mid-upper arm circumference.
