# Supplementary material for: Fortified balanced energy–protein supplementation during pregnancy and lactation and infant growth in rural Burkina Faso: A 2 × 2 factorial individually randomized controlled trial
Source: PLoS Med. 2023 Feb 6;20(2):e1004186. doi: 10.1371/journal.pmed.1004186 (PMC9943012; doi:10.1371/journal.pmed.1004186)
Supplement: S9 Table — (DOCX) [file pmed.1004186.s010.docx]

**Table S9. Subgroup analysis of the efficacy of maternal prenatal BEP supplementation on infant length-for-age z score at 6 months^1^**

| **Subgroup factor** |  | **Control** |  |  | **Intervention** | **Unadjusted difference**  **(95% CI)** | ***p*** | **Adjusted difference**  **(95% CI)** | ***p*** |
| --- | --- | --- | --- | --- | --- | --- | --- | --- | --- |
|  | ***n*** | **Mean ± SD** |  | n | **Mean ± SD** |  |  |  |  |
| Maternal age |  |  |  |  |  |  | 0.332 |  | 0.290 |
| <20 years | 150 | -0.47 ± 1.05 |  | 156 | -0.37 ± 1.00 |  |  |  |  |
| ≥20 years | 603 | -0.74 ± 1.06 |  | 553 | -0.52 ± 1.03 |  |  |  |  |
| Child sex |  |  |  |  |  |  | 0.100 |  | 0.050 |
| Male | 355 | -0.56 ± 1.13 |  | 354 | -0.50 ± 0.99 | 0.03 (-0.12, 0.19) | 0.685 | 0.00 (-0.15, 0.16) | 0.967 |
| Female | 398 | -0.50 ± 1.00 |  | 355 | -0.30 ± 1.02 | 0.22 (0.07, 0.36) | 0.004 | 0.20 (0.07, 0.34) | 0.004 |
| Maternal hemoglobin level |  |  |  |  |  |  | 0.177 |  | 0.084 |
| ≥11 g/dL | 485 | -0.52 ± 1.06 |  | 450 | -0.35 ± 1.02 | 0.18 (0.05, 0.32) | 0.007 | 0.17 (0.04, 0.30) | 0.010 |
| <11 g/dL (anemic) | 268 | -0.55 ± 1.07 |  | 259 | -0.50 ± 0.98 | 0.01 (-0.17, 0.19) | 0.917 | -0.04 (-0.22, 0.13) | 0.649 |
| Maternal BMI |  |  |  |  |  |  | 0.935 |  | 0.804 |
| ≥18.5 kg/m^2^ | 702 | -0.51 ± 1.07 |  | 662 | -0.39 ± 1.01 |  |  |  |  |
| <18.5 kg/m^2^ (underweight) | 50 | -0,70 ± 0.91 |  | 47 | -0.54 ± 0.96 |  |  |  |  |
| Maternal MUAC |  |  |  |  |  |  | 0.560 |  | 0.486 |
| ≥23 cm | 342 | -0.38 ± 1.06 |  | 306 | -0.30 ± 1.01 |  |  |  |  |
| <23 cm | 411 | -0.65 ± 1.05 |  | 403 | -0.50 ± 1.00 |  |  |  |  |
| Maternal height |  |  |  |  |  |  | 0.875 |  | 0.821 |
| ≥155 cm | 681 | -0.46 ± 1.04 |  | 645 | -0.34 ± 1.00 |  |  |  |  |
| <155 cm | 72 | -1.15 ± 1.10 |  | 64 | -1.02 ± 0.90 |  |  |  |  |
| Primiparity |  |  |  |  |  |  | 0.355 |  | 0.339 |
| No | 606 | -0.46 ± 1.02 |  | 554 | -0.36 ± 1.00 |  |  |  |  |
| Yes | 147 | -0.79 ± 1.17 |  | 155 | -0.55 ± 1.04 |  |  |  |  |
| Maternal depression possible |  |  |  |  |  |  | 0.680 |  | 0.918 |
| No | 736 | -0.53 ± 1.05 |  | 698 | -0.40 ± 1.01 |  |  |  |  |
| Yes | 17 | -0.63 ± 1.56 |  | 11 | -0.56 ± 0.69 |  |  |  |  |
| Maternal depression probable |  |  |  |  |  |  | 0.579 |  | 0.420 |
| No | 693 | -0.52 ± 1.07 |  | 658 | -0.39 ± 1.01 |  |  |  |  |
| Yes | 60 | -0.60 ± 0.95 |  | 51 | -0.59 ± 1.00 |  |  |  |  |
| Inter-pregnancy interval |  |  |  |  |  |  | 0.628 |  | 0.620 |
| ≥18 months | 739 | -0.53 ± 1.07 |  | 685 | -0.40 ± 1.01 |  |  |  |  |
| <18 months | 14 | -0.33 ± 0.82 |  | 24 | -0.30 ± 0.90 |  |  |  |  |
| Season of delivery |  |  |  |  |  |  | 0.222 |  | 0.080 |
| Plenty | 523 | -0.55 ± 1.07 |  | 471 | -0.38 ± 1.02 | 0.18 (0.05, 0.31) | 0.008 | 0.17 (0.05, 0.30) | 0.006 |
| Lean (June – September) | 230 | -0.47 ±1.04 |  | 238 | -0.44 ± 0.99 | 0.04 (-0.15, 0.23) | 0.692 | -0.01 (-0.19, 0.16) | 0.875 |
| Household food security |  |  |  |  |  |  | 0.933 |  | 0.440 |
| Food secure | 337 | -0.49 ± 1.04 |  | 317 | -0.37 ± 0.99 |  |  |  |  |
| Food insecure | 416 | -0.56 ± 1.08 |  | 392 | -0.43 ± 1.02 |  |  |  |  |

^1^Linear regression models were fitted to test interaction between prenatal BEP supplementation group and a subgroup factor with subgroup analysis considered when there was statistical significant interaction at *P*<0.10. Unadjusted and adjusted group differences were estimated using models containing allocation to the postnatal BEP supplementation, and health center and randomization block as fixed effect to account for clustering by the study design. Adjusted models additionally contained *a priori* determined set of maternal prognostic factors such as age, parity, gestational age, height, mid-upper arm circumference, body mass index and hemoglobin level at study enrolment. BEP, Balanced Energy-Protein Supplementation; BMI, body mass index; MUAC, mid-upper arm circumference. BEP, Balanced protein-energy supplement.
