# Supplementary material for: Fortified balanced energy–protein supplementation during pregnancy and lactation and infant growth in rural Burkina Faso: A 2 × 2 factorial individually randomized controlled trial
Source: PLoS Med. 2023 Feb 6;20(2):e1004186. doi: 10.1371/journal.pmed.1004186 (PMC9943012; doi:10.1371/journal.pmed.1004186)
Supplement: S1 Fig — BEP, balanced energy–protein supplementation; GA, gestational age; IFA, iron–folic acid tablets; MISAME, MIcronutriments pour la SAnté de la Mère et de l’Enfant. (DOCX) [file pmed.1004186.s011.docx]

58 women not meeting eligibility criteria:

30 women with GA ≥21 weeks at inclusion

28 multifetal pregnancies

Infants assessed at 6 mo (*n* = 753)

Subsample of infants assessed at 9 mo (*n* = 695)

Subsample of infants assessed at 12 mo (*n* = 501)

x

Infants assessed at 6 mo (*n* = 709)

Subsample of infants assessed at 9 mo (*n* = 650)

Subsample of infants assessed at 12 mo (*n* = 454)

## **Analysis**

51 women not meeting eligibility criteria:

29 women with GA ≥21 weeks at inclusion

22 multifetal pregnancies

## **Exclusion**

960 women in prenatal control group: received IFA

937 women in prenatal intervention group: received BEP + IFA

937)

2016 women assessed for eligibility

(N=2016)

119 women excluded for not meeting the inclusion criteria:

110 non-confirmed pregnancies

9 women declined to participate

## **Allocation**

1897 randomised

## **Enrolment**

22 lost to follow up during prenatal phase:

16 women left study area

6 women refused to continue

37 fetal losses and stillbirths

850 women with birth outcome data

58 lost to follow up during postnatal phase:

10 left study area

15 child death

12 refusal

21 unknown reason

39 missing data at 6 mo

27 lost to follow-up during prenatal phase:

21 women left study area

6 women refused to continue

43 fetal losses and stillbirths

809 women with birth outcome data

60 lost to follow-up during postnatal phase:

9 left study area

15 child death

7 refusal

29 unknown reason

40 missing data at 6 mo

## **Follow-up**

**Fig S1. Trial flowchart of the MISAME-III study by the prenatal intervention arms**. BEP, balanced energy-protein supplementation; GA, gestational age; IFA, iron folic acid tablets; MISAME, MIcronutriments pour la SAnté de la Mère et de l’Enfant
