# Supplementary material for: Fortified balanced energy–protein supplementation during pregnancy and lactation and infant growth in rural Burkina Faso: A 2 × 2 factorial individually randomized controlled trial
Source: PLoS Med. 2023 Feb 6;20(2):e1004186. doi: 10.1371/journal.pmed.1004186 (PMC9943012; doi:10.1371/journal.pmed.1004186)
Supplement: S2 Fig — Line graphs represent locally weighted scatterplot smoothing of observed values. Group differences were estimated using mixed-effects models with random intercepts for infant and random slopes for intervention time, with fixed effects including time, quadratic time, intervention group, and time × group interaction adjusted for clustering indicators (health center and randomization block), and a priori determined prognostic factors (maternal height, BMI, MUAC, hemoglobin, age and gestational age at inclusion, and parity). Compared to the control group, effect sizes in the prenatal only supplementation group (ES: 0.005 SD/month, 95% CI: −0.007 to 0.017, p = 0.416), the postnatal only supplementation group (ES: 0.012 SD/month, 95% CI: 0.000 to 0.024, p = 0.055), and the combined pre- and postnatal supplementation group (ES: 0.011 SD/month, 95% CI: 0.000 to 0.023, p = 0.060). BEP, balanced energy–protein supplement; BMI, body mass index; CI, confidence interval; ES, effect size (regression coefficient); IFA, iron–folic acid; LAZ, length-for-age Z-score; MUAC, mid-upper arm circumference; SD, standard deviation. (DOCX) [file pmed.1004186.s012.docx]

**
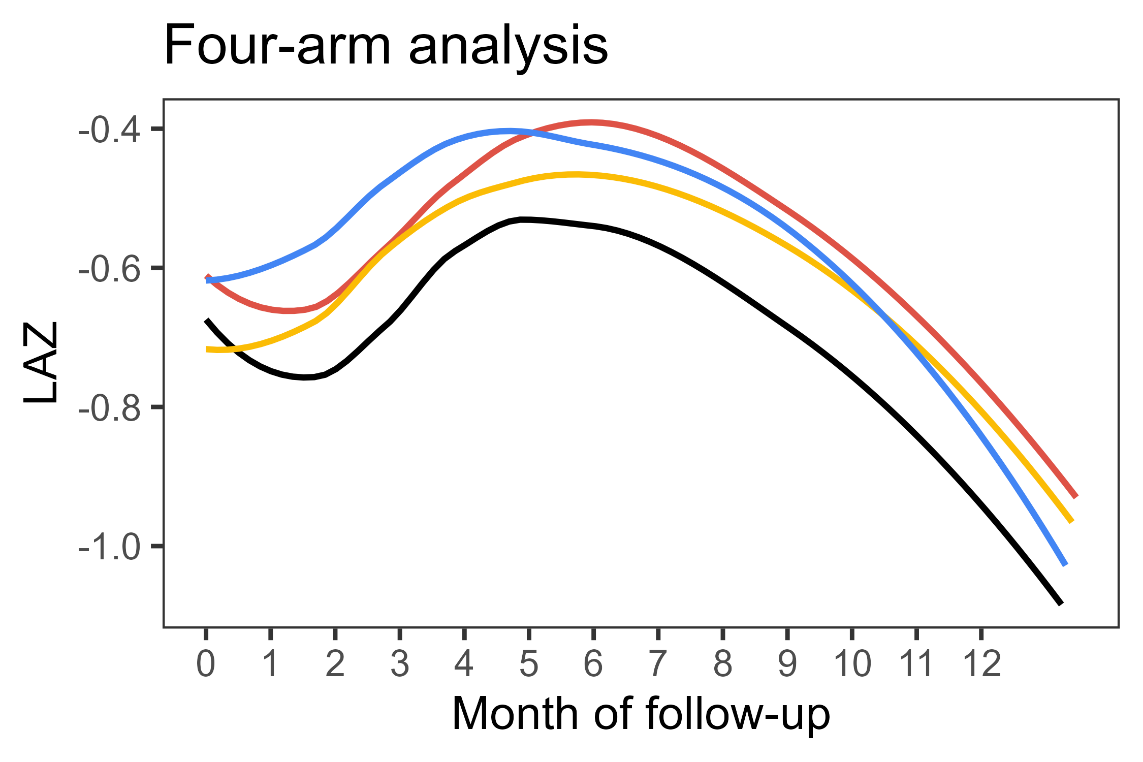
**

**Fig S2. Monthly changes in LAZ by four-arm groups including the control group receiving IFA/IFA supplementation (black line), the prenatal only supplementation group receiving BEP/IFA (blue line), the postnatal only supplementation group receiving IFA/BEP (yellow line), and the combined pre- and post-natal supplementation group receiving BEP/BEP (red line).** Line graphs represent locally weighted scatterplot smoothing of observed values. Group differences were estimated using mixed-effects models with random intercepts for infant and random slopes for intervention time, with fixed effects including time, quadratic time, intervention group, and time×group interaction adjusted for clustering indicators (health center and randomization block), and *a priori* determined prognostic factors (maternal height, body mass index, mid-upper arm circumference, hemoglobin, age and gestational age at inclusion, and parity). Compared to the control group, effect sizes in the prenatal only supplementation group (ES: 0.005 SD/month, 95% CI: -0.007 to 0.017, *p* = 0.416), the postnatal only supplementation group (ES: 0.012 SD/month, 95% CI: 0.000 to 0.024, *p* = 0.055), and the combined pre- and post-natal supplementation group (ES: 0.011 SD/month, 95% CI: 0.000 to 0.023, *p* = 0.060). BEP, balanced energy-protein supplement; CI, confidence interval; ES, effect size (regression coefficient); IFA, iron-folic acid; LAZ, length-for-age Z-score.
