# Supplementary material for: Fortified balanced energy–protein supplementation during pregnancy and lactation and infant growth in rural Burkina Faso: A 2 × 2 factorial individually randomized controlled trial
Source: PLoS Med. 2023 Feb 6;20(2):e1004186. doi: 10.1371/journal.pmed.1004186 (PMC9943012; doi:10.1371/journal.pmed.1004186)
Supplement: S1 Statistical Analysis plan — (PDF) [file pmed.1004186.s014.pdf]

# Statistical analysis plan: Impact of a prenatal and postnatal balanced energy protein supplement on birth size and post-natal child growth in Burkina Faso

Version 1.0:

Date: October 24, 2019

Cite as: MISAME study team (2019). Statistical plan MISAME-III Study, version 1, October 2019.

## Background

Pregnancy remains a challenging period in the life of many women in low- and middle-income countries. Maternal mortality remains high and many newborns suffer from premature delivery and /or gestational growth retardation both in linear and ponderal growth. Newborns born too small or too soon have a significantly higher mortality risk.

The MISAME-III (Micronutriments et Santé de la Mère et de l'Enfant) trial is a randomized controlled 2x2 factorial efficacy trial designed to assess the impact of prenatal and postnatal balanced energy-protein (BEP) supplements on fetal and early child growth in rural Burkina Faso. The nutritional composition of the BEP supplement was established during an expert convening at the BMGF in September 2016. Private sector partners prepare the supplements in the selected forms with the recommended nutrient composition. During a previous first and second phase of formative research the acceptability of several BEP supplements was evaluated in different samples of pregnant women in Burkina Faso. Four sub-studies are nested in the RCT: i) the impact of BEP on maternal and child body composition assessed using double labeled water; ii) the impact of the BEP supplement on dietary intake of pregnant women; iii) impact of BEP on breastmilk composition at delivery, 1-2 and 3-4 months of the infant's age; iv) the residual impact of BEP on child anthropometry at 9 and 12 months of age.

## Study objectives

The primary objectives of this study are that providing:

- a prenatal balanced energy-protein supplement (BEP) leads to a smaller incidence of small-for-gestational age (SGA) relative to a control group;
- a postnatal maternal balanced energy-protein supplement will improve growth in length of their offspring by the age of 6 months relative to a control group.

## Study Methods

### Study area and population

The trial will be conducted in the health district of Hounde, Hauts-Bassins region, Burkina Faso. In this health district we selected purposively 6 health center catchment areas and within these catchment areas a number of villages. We selected villages based on size, accessibility and distance to the health center to ensure that women can deliver in the health center. The study population for this trial consists of women between 15-40 years of age, residing in the study site and who become pregnant over a 12-month inclusion period.

# Study design

## Prenatal intervention

The study is organized as a community-based, individually randomized efficacy trial assessing the effect of prenatal BEP on birth outcomes.

## Postnatal intervention

Women participating in the prenatal intervention study are also randomized a second time to either receive BEP/IFA for 6 months or IFA for 6 weeks (according to national policy) after delivery. A 2x2 factorial design is thus used to assess the impact of the postnatal intervention on postnatal child and maternal outcomes (**Table 1**). A priori we consider to conduct a factorial analysis (i.e. analyzing the data by study arm) to assess the impact of the postnatal intervention on postnatal outcomes. However, such analysis relies strongly on the assumption that there is no interaction between the pre- and postnatal intervention. Such an assumption seems difficult to make given the hypothesized impacts of the individual interventions and their sequential nature. It is well documented that the presence of an interaction between interventions leads to biased main effects.[1] Therefore, the method of analysis to answer the research questions is conditional on the presence or absence of an interaction between the prenatal and postnatal intervention. As a consequence, a two-step approach is required to determine the analysis strategy. Step one of the analysis entails the testing of the interaction between the prenatal and postnatal intervention. Depending on the outcomes of step one, data will be either analyzed stratified by prenatal and postnatal intervention (factorial analysis) or analyzed stratified by pre-and postnatal intervention and their combinations (i.e. 4 groups) under step two. In the latter case, we make a clear distinction into the level of importance (primary or secondary) of the hypotheses to test as shown in **Table 2**.

**Table 1 Schematic overview of 2 x 2 factorial study design to assess the impact of BEP on postnatal study outcomes**

| | | Postnatal intervention | |
|---|---|---|---|
| | | BEP/IFA | IFA |
| Prenatal intervention | BEP/IFA | AB | B |
| | IFA | A | C |

**Table 2 Planned hypothesis tests according to priority in the absence or presence of an interaction between prenatal and postnatal intervention.**

| Hypothesis tests | Scenario 1: Absence of interaction | Scenario 2: Presence of interaction |
|---|---|---|
| (AB+A) vs. (B+C) | Primary endpoint analysis | Not possible |
| (AB+B) vs. (A+C) | Secondary analysis | Not possible |
| AB vs. C | Secondary analysis | Primary endpoint analysis |
| A vs. C | Secondary analysis | Primary endpoint analysis |
| B vs. C | Secondary analysis | Secondary analysis |

In the absence of an interaction between the prenatal and postnatal intervention, we plan to conduct a factorial analysis comparing the impact of postnatal BEP/IFA (AB+A) to postnatal IFA (B+C) on child LAZ at 6 months of age (and other secondary outcomes). A secondary analysis would be to assess the impact of prenatal BEP/IFA (AB+B) relative to prenatal IFA (A+C) on the same outcome. Further exploratory analyses can compare the impact of combined AB, A and B to the control group (C) who received prenatal and postnatal IFA.

[1] Boris Freidlin and Edward L. Korn, 'Two-by-Two Factorial Cancer Treatment Trials: Is Sufficient Attention Being Paid to Possible Interactions?', *Journal of the National Cancer Institute*, 109.9 (2017), 1–5; Edward L. Korn and Boris Freidlin, 'Non-Factorial Analyses of Two-by-Two Factorial Trial Designs', *Clinical Trials*, 13.6 (2016), 651–59.

In the presence of an interaction between the prenatal and postnatal intervention, the primary analysis consist of testing the combined prenatal and postnatal BEP/IFA relative to the control group (pre-and postnatal IFA) and to test the postnatal BEP/IFA (A) versus the control group C. Additional exploratory analyses include the assessment of the impact of prenatal BEP/IFA (B) relative to the control group (C) on postnatal maternal and child outcomes.

## Study eligibility and recruitment

A census will be conducted in a selection of villages belonging to 6 health center catchment areas. These villages were selected based on their year-round accessibility and their distance to the health center (<7 km). The census will identify women in the reproductive age range (here defined as 15 to 40 years). To identify pregnancy early, community workers (CW; recruited by the study) will visit women of reproductive age every five weeks. With a population of 9,700 women living in selected villages of 6 health center catchment areas to follow-up to detect a pregnancy and 60-70 women per community health worker, 142 CW will be recruited. Based on delivery statistics provided by the local health authorities, the villages belonging to the 6 catchment areas provide 2,125 live births per year. In order to achieve the total sample size of 1,776 live births, one will need to follow up the 9,700 women in the reproductive age group during ±10 months, which corresponds to the enrollment phase of the study.
Study inclusion criteria are:

- Women 15-40 years of age at study inclusion;
- Pregnant as determined by a pregnancy test and confirmed by ultrasound;
- Women who signed the IC form (in case of minors, the parents or husband sign).

Study exclusion criteria:

- Pregnant women planning to leave the area before delivery
- Pregnant women who plan to deliver outside the area
- Pregnancies with a gestational age >20 weeks at study inclusion;
- Women with multifetal gestations (exclusion from analysis);
- Women who are allergic to peanuts.

Consenting women will be visited every five weeks by a CW who will assess the date of the last menstrual period(LMP). If LMP was more than 5 weeks before the visit, the CW will refer the candidate pregnant woman to the nearest health center for a pregnancy test. Trial midwifes will confirm the pregnancy using a dipstick pregnancy test. Pregnant women will receive an ultrasound to assess gestational age at inclusion. Using a pre-established randomization scheme, consenting women will be allocated to either the intervention or the control group using sealed envelopes. CWs will be tasked to visit pregnant and lactating women daily to provide the intervention or control supplements. CWs will be equipped with cell phones and given a free number to communicate with the health center staff and the field officers. Adherence to the visits at the health center will be followed up by the CW.

## Study intervention

Women allocated to the prenatal intervention group will receive daily BEP and a iron/folic acid tablet (60mg iron and 400mcg Folic acid) until delivery. Women allocated to the control group will receive a daily iron/folic acid tablet (60mg iron and 400mcg Folic acid) until delivery. Mothers allocated to the postnatal intervention group will be given daily BEP and iron and folic acid tablets until 3 weeks after delivery. From that moment onwards lactating women will be visited weekly to receive a weekly dose of BEP until the child reaches 6 months age. Women allocated to the postnatal control group will be given daily iron and folic acid tablets until the child reaches the age of 6 weeks. After 6 weeks, control mothers will receive weekly visits to monitor maternal and child health. By maintaining the same schedule of home visits for both study groups, the study prevents any selective influence from the CW home visits themselves.

## Study ethics

The study protocol is approved by the ethics committee of the Ghent University hospital in Belgium and the ethics committee of the Centre Muraz in Bobo-Dioulasso, Burkina Faso. The study is registered on clinicaltrials.gov (identifier NCT03533712). Written informed consent will be sought and obtained before including any participant in the study.

# Controls

## Level of blinding

The allocation to intervention or control study group is not blinded from the participants. However, outcome assessors will be blinded to the extent possible. The distribution of the study supplements happens during home visits in the villages, while outcome assessment is done at the health center (antenatal consultation, delivery, post-natal growth monitoring). Primary study outcome assessors also have no role in the distribution of the supplements.

## Method of treatment assignment/randomization

At study inclusion, eligible pregnant women will be randomly assigned to the prenatal intervention or control group, and randomly assigned to a postnatal intervention or control group.
We will apply a stratified permuted block randomization schedule to allocate women to the prenatal intervention or control group and in a next step to allocate women to a postnatal intervention and control group. Per health center (=stratum) women will be individually randomly in permuted blocks of 8 so that per block equal numbers are obtained in the prenatal intervention (n=4) and control (n=4) group and equal numbers are also obtained in the postnatal intervention (n=4) and control (n=4) group. The double random sequence will be generated before the start of the study using Stata 15.1 (Statacorp, Texas) by a research analyst (F.B.) who is not part of the study team and who is unaware of the study's objectives and procedures. A time coded Stata log-file is available upon request after the completion of the study to assess the randomization sequence generation.

The pre- and postnatal allocation groups codes are placed in a sealed opaque envelope by a person not participating in the implementation of the trial. When allocating a participant, the trial midwife will open the next sealed envelope and transmit the assignment codes (for both prenatal and postnatal supplements) and personal identifier of the study participant to the responsible of the supplement distribution. The person responsible of the supplementation logistics delivers the supplements to the CW.

# Study outcomes

The primary study outcomes of the efficacy trial are:

- Incidence of SGA (defined as a birth weight <10th centile intergrowth 21$^{st}$ reference)
- Length-for-age Z-score (LAZ; calculated using the WHO growth reference) in children at 6 months of age

Secondary study outcomes on maternal, newborn and child level are shown in **table 3**

**Table 3 Secondary study outcomes at maternal, newborn and child level**

| Maternal level | Newborn level | Child level |
|---|---|---|
| Prenatal weight gain (weight change between study inclusion until just before delivery): total and trimester-specific | Birth weight (measured within 72[2] hours after birth) | Weight-for-Age Z-score at 6 months of age (WAZ; calculated using the WHO growth reference) |
| Gestational weight change (defined as the difference in maternal weight between maternal weight one month after delivery and maternal weight at study inclusion) | Birth length (measured within 72[3] hours after birth) | Weight-for-Length Z-score at 6 months of age (WLZ; calculated using the WHO growth reference) |
| Probable and possible maternal post-natal depression (EPDS 12/13 and EPDS 9/10 respectively) at 2 months of child age | Ponderal or Rohrer's index at birth (birth weight/birth length$^3$) | Stunting (LAZ<-2) at 6 months of age |
| Probable and possible maternal post-natal depression (EPDS 12/13 and EPDS 9/10 respectively) at 6 months of child age | Incidence of preterm birth (<37 weeks of gestation) | Wasting (WLZ<-2) at 6 months of age |
| Women's mean dietary diversity score (measured biweekly) using the 10 food group indicator[4] | Gestational age at delivery | Underweight (WAZ<-2) at 6 months of age |
| Women's minimum dietary diversity dietary diversity score (measured biweekly) | Large-for-gestational age (defined as a birth weight ≥90th centile intergrowth 21st reference) | Duration of exclusive breastfeeding during the first 6 months of age. |
| Maternal anemia at the third antenatal consultation[5] | Chest circumference (measured within 72 hours after birth) | Incidence of child wasting over first 6 months of life |
| | Head circumference (measured within 72 hours after birth) | Weight gain over first 6 months of life |
| | Arm circumference (measured within 72 hours after birth) | Child mortality (between birth and 6 months of age) |
| | Fetal loss (fetal death at <24 completed weeks of gestational age) | Monthly change in LAZ over first 6 months of life |
| | Stillbirths (fetal death at ≥28 weeks gestational age) | Monthly change in WHZ over first 6 months of life |
| | Neonatal mortality (deaths between birth and ≤28 days of life) | Monthly change in WAZ over first 6 months of life |
| | Early neonatal mortality (deaths between birth an ≤ 7 days of life | Monthly change in head circumference over first 6 months of life |
| | Late neonatal mortality (deaths between >7 days and ≤28 days of life) | Child morbidity symptoms (longitudinal prevalence over first 6 months of life); signs include fever, vomiting, diarrhea, cough, difficult breathing, running nose. |
| | | Anemia (hemoglobin concentration <11g/dL) at 6 months of age |
| | | Hemoglobin concentration at 6 months of age |

- *Sub-study 1: child and maternal body composition*

[2] In practice we aim at measuring all birth anthropometry within 24h after delivery.

[4] Yves Martin-Prevel and others, *Moving Forward on Choosing a Standard Operational Indicator of Women's Dietary Diversity* (Rome: FAO, 2015).

[5] Anemia is defined as a hemoglobin concentration below 10.5 g/dL in the second trimester and below 11.0 g/dL during the third trimester to account for plasma expansion

In a subsample of mother-child dyads we will assess the impact of the prenatal intervention on neonatal and maternal fat mass index (fat mass/ length$^2$) and fat-free mass index (fat-free mass/ length$^2$) by collecting saliva samples that will be analyzed by a portable Fourier Transform Infrared reader (Agilent FTIR 4500 series).

- *Sub-study 2: dietary assessment of pregnant women*

A dietary assessment study will be conducted in a cross-sectional sub-sample of pregnant women to assess the impact of the prenatal dietary intervention on the dietary intake. The main objective of this sub-study is to assess to what extent the usual dietary intake is substituted with BEP supplement as compared to the control group. More specifically, following study outcomes will be assessed:

- Daily energy and nutrient intakes
- Mean probability of Adequacy calculated based on 11 micronutrients of the diet without accounting for the BEP
- Women's dietary diversity score (10 food groups)
- Minimum Dietary Diversity for Women (MDD-W) (5 out of 10 food groups)[6]

The West African food composition table will be used to convert food intake to energy and nutrient intake

- *Sub-study 3: breast milk composition*

A separate analysis plan will be prepared by the principal investigators of this sub-study

- *Sub-study 4: impact of prenatal and postnatal intervention on child anthropometry in a subsample of 6-12 months*

In about half the sample of early enrolled mothers we will conduct follow-up visits at 9 and 12 months of age to assess the impact of pre- and postnatal intervention on the monthly change of LAZ, WAZ, WLZ, and head circumference of their children. We will also assess the impact of the interventions on the prevalence of stunting at 9 and 12 months of age and the incidence of wasting and acute malnutrition (defined as WLZ<-2 or MUAC<125mm or nutritional edema).

# Sample size

**Main trial**

- **Small-for-gestational age:** SGA estimates are based on the MISAME-II study outcomes. With an SGA prevalence of 32% and an anticipated decrease of 7%, a sample size of 652 subjects per arm is needed with α=0.05 and β=0.20. In the previous MISAME studies[7], a ~26% loss of information by a combination of abortions, still births, multifetal pregnancies, out-migrations, maternal deaths and incomplete data occurred. The sample size needs to increase to 888 (total 1,776 subjects) subjects per arm to accommodate for these possible losses.
- **Length-for age Z-score at 6 months of age:**
  - <u>In the absence of a statistically significant interaction between pre-and postnatal intervention:</u> To detect a difference of 0.18 Z-score (SD=1.1[8]) between study arms with α=0.05 and β=0.20, a sample of 588 children per study arm (1,176 children in total). This implies that if ~1400 singleton live births are available, we allow for a maximum lost to follow-up of 16%.
  - <u>In the presence of a statistically significant interaction between pre-and postnatal intervention:</u> A total sample size of 1,176 represents 294 children per factorial combination of the prenatal and postnatal study group (4 groups in total). A subgroup size of 294 allows to detect a difference in LAZ at 6 months of child age of 0.28 assuming a SD of 1.1, α=0.025 (Bonferroni correction for two primary endpoint analyses and β=0.2.

---

[6] Yves Martin-Prevel and others, *Moving Forward on Choosing a Standard Operational Indicator of Women's Dietary Diversity* (Rome: FAO, 2015).

[7] Lieven Huybregts and others, 'Prenatal Food Supplementation Fortified with Multiple Micronutrients Increases Birth Length: A Randomized Controlled Trial in Rural Burkina Faso.', *The American Journal of Clinical Nutrition*, 90.6 (2009), 1593–1600; Dominique Roberfroid and others, 'Effects of Maternal Multiple Micronutrient Supplementation on Fetal Growth: A Double-Blind Randomized Controlled Trial in Rural Burkina Faso.', *The American Journal of Clinical Nutrition*, 88.5 (2008), 1330–40.

[8] Estimated from a subgroup of 6-6.9 months old children from cross-sectional survey conducted in the Gourcy health district (Becquey et al, PloS Medicine 2019)

## Sub-studies

1. *Impact of the intervention on neonatal body composition and maternal body composition*

We calculate that a sample size of 440 neonates per study arm (880 in total) would allow to detect an effect size of 0.2 on a main effect on the fat mass index (fat mass/length$^2$) and 0.4 for an interaction effect between supplementation and low vs. high BMI or MUAC at study inclusion, with a type I error of 5% and a statistical power of 80% assuming 10% of the samples would present unreadable results.
Furthermore, we calculate that a sample size of 195 women per study arm (n=390) would allow to detect an effect size of 0.3 on the fat mass index (fat mass/length$^2$) with $\alpha=0.05$ and $\beta=0.20$, assuming 10% of the samples would present unreadable results. The 390 women will be selected at random from the 880 children providing saliva samples for neonatal body composition

2. *Impact of the intervention on dietary intake:*

We calculate that 242 women per study arm (484 in total) need to be included in order to detect a minimal difference of half the energy dose provided the BEP (194kcal) with a mean energy intake of 2050 kcal (SD: 643kcal)[9] between study arms, with $\alpha=0.05$ and $\beta=0.90$ and 10% non-response.

3. *Impact of the intervention on breast milk composition*

We refer to the protocol of the breastmilk sub-study

4. *Impact of the intervention on child anthropometry in a subsample of children 9 and 12 months of age*

No formal sample size calculation can be done because the sample size depends on the duration of the study. The study will be finalized once all children reach the age of 6 months. By that time an unknown proportion of children (around half of the sample) will have reached the age of 9 or 12 months and will thus constitute the subsample.

# Efficacy analyses of the prenatal intervention

## Main trial

An individually randomized controlled study design is used to assess the effect of the prenatal intervention on newborn and maternal study outcomes.

The primary study outcome analysis will be by intention-to-treat, i.e. outcomes for all study outcomes will be analyzed as randomized and according to pre-specified eligibility criteria for analysis.

Unless specified otherwise, continuous outcomes measured at one time point will be analyzed using ordinary least squares regression. For binary outcomes measured at one timepoint, we will calculate risk ratios that will be analyzed using log-binomial regression models. In the unlikely event that the latter models do not converge, Poisson regression models with robust estimation of standard errors will be used.

All birth weights will be measured at least in duplicate within 72 hours, although we aim at collecting all birth anthropometry within 24 hours after delivery. Birth weights measured after 72 hours will not be considered in the primary outcome analysis.

The impact of the prenatal intervention on gestational weight gain will be assessed by testing the linear interaction product between gestational age and the prenatal intervention allocation. For this analysis a mixed-effects regression model will be used with *mother* as a random intercept and *gestational age* as a random slope. We will compare the fit between a linear mixed effects model (model 1) and an identical model but with the addition of a quadratic term of gestational age (model 2). The choice between model 1 and 2 will be decided based using likelihood ratio test. The impact of the intervention on maternal hemoglobin concentration and anemia during the third antenatal consultation will be adjusted for gestational age at the measurement

[9] Huybregts, Lieven (2010) Prevention of intrauterine growth retardation by food supplementation in rural Burkina Faso. Ghent, Belgium

All primary analyses will be adjusted for prognostic covariates (listed in **table 4**) that differ at baseline between study groups for more than 2.5% in absolute value. These factors were previously reported to be associated with birth size. This adjustment strategy will account for baseline imbalances and will increase statistical power by reducing variance. All analyses will be adjusted for health center (fixed effect) to reflect the stratified sampling design. All statistical tests will be two-sided, at the 5% level of significance. Where more than 10% of observations are missing for a dependent variable (not counting non-eligibility), we will report the number of observations used in the analysis.

**Table 4. Prognostic factors of study outcomes at birth and during childhood**

| **Factors** |
|---|
| Maternal height at study inclusion |
| Maternal BMI at study inclusion |
| Maternal MUAC at study inclusion |
| Maternal hemoglobin concentration at study inclusion |
| Maternal age at study inclusion |
| Gestational age at study inclusion |
| Primiparity |

## Efficacy analyses of the postnatal intervention

As mentioned in the section of the ‘Study design’, a two-step approach will be taken to conduct the efficacy analysis of the postnatal intervention. Step one of the analysis entails the testing of the interaction between the prenatal and postnatal intervention. If a statistically significant interaction between the pre-and postnatal intervention is found (at 5% level), the analysis will be conducted stratified by pre- and postnatal intervention and their combinations (i.e. 4 groups). As explained in the section of the study design, in such case there are two primary endpoint analyses. To account for multiple testing a Bonferroni adjustment is adopted, therefore the type I error (or α) for two primary endpoint analyses is set at 2.5% (5%/2 tests). If there is no evidence of an interaction between pre- and postnatal intervention, we will conduct a factorial analysis where the primary endpoint analysis entails testing the effect of the postnatal intervention, adjusting the regression model for the prenatal intervention.

The primary study outcome analysis will be by intention-to-treat (ITT), i.e. outcomes for all study outcomes will be analyzed as randomized and according to pre-specified eligibility criteria for analysis.

Unless specified otherwise, continuous outcomes measured at one time point will be analyzed using ordinary least squares regression. For binary outcomes measured at one timepoint, we will calculate risk ratios that will be analyzed using log-binomial regression models. In the unlikely event that the latter models do not converge, Poisson regression models with robust estimation of standard errors will be used. All analyses will be adjusted for health center (fixed effect) to reflect the sampling design.

The impact of the postnatal intervention on the change in LAZ, WLZ, WAZ, MUAC and head circumference will be assessed by testing the linear interaction product between child age and the postnatal intervention allocation. For this analysis a mixed-effects regression model will be used with *child* as a random intercept and *child age* as a random slope. We will compare the fit between a linear mixed effects model (model 1) and an identical model but with the addition of a quadratic term of gestational age (model 2). The choice between model 1 and 2 will be decided using likelihood ratio test. For outcomes like WLZ and MUAC that are known to vary more by season, we will first inspect if there exists a linear or quadratic relationship with child age. If not, we will fit a restricted cubic spline regression model with knots set at local maxima/minima of the first derivative function calculated from a kernel-weighted local polynomial model that fits the study outcome as a function of child age. For the analysis of the subsample of children for which we will have data at 9 and 12 months of age, a similar approach will be taken.

All statistical tests will be two-sided, at the 5% level of significance. Where more than 10% of observations are missing for a dependent variable, we will report the number of observations used in the analysis.

# Considerations for analysis

## Baseline characteristics

For all variables measured, the available values at enrollment, i.e. before the start of the intervention, will be considered as baseline characteristics. The background characteristics of subjects who completed the study will be presented by prenatal and postnatal treatment group. Following CONSORT guidelines, no statistical tests will be conducted comparing baseline characteristics between study arms. Differences in baseline characteristics will be appreciated by comparing values of means and proportions.

The presentation of baseline characteristics will be done as follows:

- Categorical variables: frequencies and percentages, as appropriate. Percentages will be calculated based on the number of participants for whom data are available.
- Continuous variables: mean and SD or median and interquartile range, as appropriate.
- Where data for certain participants are missing, the number of participants included in the analysis will be indicated.

**Table 5 gives an overview of the characteristics at study enrollment that will be presented by prenatal and postnatal treatment group**

| | Prenatal intervention | | Postnatal intervention | |
|---|---|---|---|---|
| **Characteristics** | Control | Intervention | Control | Intervention |
| **Household level** | | | | |
| HH food insecurity | x | x | x | x |
| WASH proxy[10] (tertiles) | x | x | x | x |
| SES proxy (tertiles) | x | x | x | x |
| Polygamous HH(%) | x | x | x | x |
| Primary economic activity of head of household (%) | x | x | x | x |
| Number of under 5 children in household (mean/SD) | x | x | x | x |
| **Parental level** | | | | |
| Head of household school attendance (None, Primary, Secondary) | x | x | x | x |
| Maternal school attendance (None, Primary, Secondary) | x | x | x | x |
| Maternal ethnic group | | | | |
| Maternal religion | | | | |
| Maternal revenue generating activities (Formal salary, small commerce, wholesale, shop, artisan, services, homestead gardening/animal breeding) | x | x | x | x |
| Maternal age(y) | x | x | x | x |
| Maternal weight(kg) | x | x | x | x |
| Maternal Height (m) | x | x | x | x |
| Maternal BMI (kg/m$^2$) | x | x | x | x |
| Maternal underweight (BMI<18.5 kg/m$^2$) | x | x | x | x |
| Maternal mid-upper arm circumference (cm) | x | x | x | x |
| Maternal subscapular skinfold | x | x | x | x |
| Maternal tricipital skinfold | x | x | x | x |
| Maternal hemoglobin (g/dL) | x | x | x | x |

[10] WASH and SES proxy scores are first components of a principal component analysis based on a set of indicators.

| | | | | |
|---|---|---|---|---|
| Maternal anemia (Hb<11 g/dl) | x | x | x | x |
| Maternal severe anemia (Hb<7 g/dl) | x | x | x | x |
| Prenatal possible depression (EPDS≥10) | x | x | x | x |
| Prenatal probable depression (EPDS ≥13) | | | | |
| Bed net use | x | x | x | x |
| **Pregnancy level at study inclusion** | | | | |
| Gestational age (wk) | x | x | x | x |
| Trimester of gestation (first, second, third) | x | x | x | x |
| Parity (0, 1-2, >=3) | x | x | x | x |
| At least one previous fetal death (%) | x | x | x | |
| Nr of previous child deaths (0, 1-2, >=3) | x | x | x | x |
| Interpregnancy interval (months) | x | x | x | x |
| **Birth characteristics** | | | | |
| Birth weight (kg) | na | na | x | x |
| Birth length (cm) | na | na | x | x |
| Rohrer Ponderal index (g/cm3) | na | na | x | x |
| Head circumference (cm) | na | na | x | x |
| Thoracic circumference (cm) | na | na | x | x |
| Arm circumference (cm) | na | na | x | x |
| Gestational age (wk) | na | na | x | x |
| LBW [n (%)] | na | na | x | x |
| SGA [n (%)] | na | na | x | x |
| Preterm delivery [n (%)] | na | na | x | x |
| Newborn sex | na | na | x | x |
| Cesarean section [n (%)] | na | na | x | x |
| Prenatal intervention (BEP/IFA vs. IFA) [n (%)] | na | na | x | x |

Na, not applicable

## Potential effect modifiers

Studying effect modification of an intervention offers important insights in the mechanism of a possible impact. In this study, effect modification will be explored for a list of covariates collected at study inclusion. Given that these tests are explorative in nature, we assess interaction terms at a statistical significance set at 10%. If there are indications of effect modification by one or more covariates, we will present impact results stratified by the(se) covariate(s).

The following variables are considered as candidate effect modifiers of the prenatal and postnatal intervention:

**- Maternal underweight at study inclusion defined as:**
1. BMI<18.5 kg/m2 (underweight status)
2. MUAC<23 cm

**- Maternal undernutrition at study inclusion defined as;**
1. Lowest BMI tertile
2. Lowest MUAC tertile

**- Maternal anemia at study inclusion defined as:**
Hb concentration<11 g/dL

**- Maternal short stature**
Height<155cm

**- Maternal age at study inclusion:**
group below 20 years
**- Maternal and head of household education**
Completed primary education
**- Prenatal possible depression**
Edinburgh depression score ≥10
**- Prenatal probable depression**
Edinburgh depression score ≥13
**- Primiparity**
Primiparity
**- Household food insecurity:**
Based on the definition provided by the HFIAS scale (Category food secure vs. food insecure)
-**WASH index**
Lowest tertile of PCA derived WASH proxy index (based on USAID WASH indicators)
- **SES index**
Lowest tertile of PCA derived SES proxy index (based on household assets and concession construction material)
- **Child sex**
- **Season of delivery; see [11]**
Lean season (June-September)
- **Gestational age at enrolment**
Lowest tertile
- **Interpregnancy Interval**
Group below 18 months

One effect modification is of particular interest. Results from the MISAME-II trial[12] in Burkina Faso comparing the effect of an LNS fortified with multiple micronutrients to multiple micronutrients tablet on birth anthropometry suggest that combining energy with micronutrients supplementation during the prenatal phase leads to a larger birth length. Interestingly, this impact on birth length was significantly larger in newborns of mothers with low early-pregnancy BMI (BMI<18.5kg/m2) or anemia, or multigravidity On the other hand, a sub-study nested in this trial found an important effect modification of the effect of LNS on cord leptin by maternal BMI and MUAC during early gestation and primigravidity[13]. According to literature, cord leptin is a reflection of the fetal fat mass (95% of cord leptin is produced by the fetal compartment). In this study, we aim to test if the impact of BEP on neonatal body composition is modified by low vs. high BMI or low vs. high MUAC, and primiparity.

## Treatment of Missing data

Missing birth outcome data will be imputed using multiple data imputation (n=500 imputations) based on chained equations. Predictors used to aid in the estimation of missing birth outcomes will be maternal height, maternal BMI at study inclusion, maternal prenatal weight gain, maternal hemoglobin concentration at study inclusion, maternal age, maternal school attendance, primiparity, child sex, household food insecurity, household WASH index, gestational age at inclusion, and month of inclusion (Fourier transformed to account for circularity).

## Treatment of inclusion and randomization errors.

Under the ITT principle women allocated to the wrong intervention or who received the wrong supplement will be analyzed according to the allocation specified by the randomization. We here specify *a priori* that

[11] Toe and others, 'Seasonality Modifies the Effect of a Lipid-Based Nutrient Supplement for Pregnant Rural Women on Birth Length', *Journal of Nutrition*, 145.3 (2015).
[12] Huybregts and others, 'Prenatal Food Supplementation Fortified with Multiple Micronutrients Increases Birth Length: A Randomized Controlled Trial in Rural Burkina Faso.', *The American Journal of Clinical Nutrition*, 90.6 (2009), 1593–1600;
[13] Huybregts and others, 'Prenatal Lipid-Based Nutrient Supplements Increase Cord Leptin Concentration in Pregnant Women from Rural Burkina Faso.' *Journal of Nutrition*, 143(5):576-83

the primary outcome analysis will only include singleton pregnancies. We will enroll multifetal pregnancies in the study to ensure acceptability of the trial by the community. We learned from our experience of the two previous MISAME trials conducted in the same setting that it is unacceptable to the community that women with multifetal pregnancies would be treated differently by the project than singleton pregnancies. Prespecifying ineligibility for analysis based on objective criteria is an acceptable practice [14]. A sensitivity analysis will be conducted including all pregnancies.

## Interim analyses and Data monitoring

No interim analysis of primary endpoints will be conducted. Adverse and possible serious adverse events (SAE) will be monitored at every contact between community-based project workers and participating pregnant and lactating women. All suspect cases will be referred to the nearest health center where the trial doctor and assistant nurse will provide a diagnosis. They will subsequently assess if the recorded morbidity is likely to be associated with the study intervention. All cases are recorded and documented. SAE are reported to the data safety and monitoring board and the Ethics committee of Centre Muraz (Bobo-Dioulasso, Burkina Faso) within a week.

### Sensitivity analysis

Following sensitivity analyses will be undertaken.

1. **Adjusted analyses on primary and secondary study outcomes.** This concerns analyses adjusted in addition for more distal prognostic factors (other than shown in **table 4**) that differ more than 2.5% at baseline. These covariates include maternal and head of household school attendance, household food insecurity, SES proxy, WASH proxy, and whether the household is polygamous.
2. ***Per protocol* analysis**: the impact of the prenatal and postnatal intervention will be assessed in a subsample of women with a compliance rate ≥75%[15]. Participants will be analysed by the pre- and postnatal supplements they received. Compliance is defined in two ways:
   a. Strict compliance (number of days with observed intake over total days of between study inclusion and delivery for the prenatal intervention and between delivery and 6 months of child age)
   b. Total compliance (as the number of days supplements were delivered -and thus available- to the participating mother over total days of between study inclusion and delivery for the prenatal intervention and between delivery and 6 months of child age).
3. Efficacy analyses will be run including multifetal pregnancies adjusting for the number of foetuses and using birth weights collected 72 hours or more after delivery[16], adjusting for the time of measurement.

[14] Lisa N. Yelland and others, 'Applying the Intention-to-Treat Principle in Practice: Guidance on Handling Randomisation Errors', *Clinical Trials*, 12.4 (2015), 418–23.
[15] In case there is less than 10% of the sample with ≥75% of compliance, the compliance cutoff will be reduced to 60% for this sensitivity analysis.
[16] Birth weights collected after 72 hr after delivery will be back calculated using a method described by Katz et al.
